# The Invisible Balance: How Prebiotics and Probiotics Can Support Fish Health and Development

**DOI:** 10.3390/life16091479

**Published:** 2026-09-04

**Authors:** Horia-Ioanid Stan, Cristian-Alin Barbacariu, Cristina Simeanu, Cristina-Gabriela Radu-Rusu, Lazăr Mircea, Daniel Simeanu

**Affiliations:** 1 Faculty of Food and Animal Sciences, “Ion Ionescu de la Brad” University of Life Sciences, 3 Mihail Sadoveanu Alley, 700489 Iasi, Romania; horia.stan@iuls.ro (H.-I.S.); cristina.radurusu@iuls.ro (C.-G.R.-R.); mircea.lazar@iuls.ro (L.M.); daniel.simeanu@iuls.ro (D.S.); 2 Research and Development Station for Aquaculture and Aquatic Ecology, “Alexandru Ioan Cuza” University, Carol I, 20A, 700505 Iasi, Romania

**Keywords:** aquaculture, functional feed additives, growth performance, feed utilization, gut microbiota, intestinal health, immune response, disease resistance, oxidative stress, water quality

## Abstract

Aquaculture is a field that has developed rapidly over the past decade with the emergence of intensive farming systems that have significantly increased global fish production. However, this intensification has also led to the emergence of diseases caused by various pathogens, often resulting in extensive antibiotic use and, consequently, antimicrobial resistance (AMR). Consequently, research has focused on finding other sustainable alternatives to avoid the overuse of antibiotics and to protect both human and animal health. Probiotics and prebiotics have been extensively studied for their wide range of effects on growth, immunity, and the control of various pathogens. Through their complex mechanisms, they can also improve water quality, mitigate the effects of various physicochemical stressors, and enhance reproduction. This review aims to present the evolution of these concepts, with an emphasis on their effects, which often vary depending on the type of additive used, the dose, and the fish species.

## 1. Introduction

Aquaculture plays an increasingly important role in ensuring the global supply of aquatic food, and in 2024, its production of aquatic animals exceeded 100 million metric tons [1]. Against the backdrop of growing demand for fish, the sector’s development must aim not only for higher production but also for efficient and sustainable systems [2]. However, as aquaculture has advanced and intensive farming systems have developed, pressures such as hypoxia, emerging diseases, and the misuse of antibiotics have emerged, which can affect fish health while simultaneously promoting antimicrobial resistance in the environment [3,4,5]. Recognizing the issues associated with these risks as aquaculture has developed, research has begun into new, sustainable, and as safe as possible methods to combat the problem of antimicrobial resistance and improve animal welfare [4,5].

Consequently, interest in probiotics and prebiotics has grown considerably, with lactic acid bacteria and those of the genus Bacillus being among the most intensively studied and subsequently the most widely used in aquaculture [6,7]. In addition, research has focused intensively on identifying new bacterial or yeast isolates from the environment or indigenous to the host in order to discover new characteristics [8,9]. Prebiotics have also been studied extensively; among the most common compounds are β-glucans, MOS, FOS, GOS, XOS, and inulin [10,11]. Both probiotics and prebiotics are valued in aquaculture because, once administered, they can act as growth promoters [6,11], modulate the gut microbiota, and support intestinal function [9,10], stimulate the immune system, and provide resistance against various pathogens [8,12,13], including tolerance to different physicochemical stressors [8,11]. In addition to these effects, certain probiotic strains can also improve water quality [6,7]. One such example is *Bacillus subtilis* strain N4, which exhibited heterotrophic nitrification and aerobic denitrification, effectively controlling nitrite accumulation in Nile tilapia farming systems [14]. However, the results in both cases are not uniform, varying depending on the probiotic and prebiotic used, the administered dose, and the fish species. The present review aimed to provide up-to-date information concerning the effects of probiotics and prebiotics separately, while synbiotics were not included in order to better distinguish the individual effects of probiotic and prebiotic administration in fish aquaculture.

## 2. Materials and Methods

The literature used in this review was identified by searching the Web of Science, Scopus, PubMed, and Google Scholar databases, and publications from 1900 to 2026 were analyzed. The literature search was conducted between January and August 2026. Older studies were used to present the origin and evolution of the concepts of probiotics and prebiotics, while information on their applications in aquaculture was drawn primarily from recent studies and reviews. The search was conducted using the terms “probiotic,” “prebiotic,” “fish,” and “aquaculture,” combined with phrases referring to sources, selection and safety criteria, mechanisms of action, growth, feed utilization, gut microbiota, immunity, resistance to pathogens, oxidative stress, reproduction, and water quality. To identify studies focused on specific probiotic microorganisms and prebiotic compounds, their names were also used as additional search terms, such as *Bacillus*, *Lactobacillus*, *Lactococcus*, *Pediococcus*, and *S. cerevisiae*, as well as inulin, FOS, MOS, and β-glucans.

Original articles, reviews, official reports, and regulatory documents that provided information on probiotics and prebiotics or on the effects of their administration in fish were included. This review was based on an analysis of 407 bibliographic sources, of which approximately 280 were experimental articles, while the remaining 127 were review articles and other documentary sources. The selection of studies was based on the relevance of the title, abstract, and full text to the topics addressed in the review. Studies that were not directly related to fish or aquaculture, studies conducted exclusively on terrestrial animals, and publications that did not allow for the separation of the effects of probiotics or prebiotics from those of other additives were excluded.

## 3. Probiotics

### 3.1. Historical Background and the Evolution of the Definition

An early milestone in the development of the concept of beneficial microorganisms was the research conducted by Henry Tissier, who, in 1899, isolated from the feces of a breastfed infant a microorganism described as “*Bacillus bifidus communis*,” now classified as *Bifidobacterium bifidum* [15,16]. Based on his experiments, Tissier suggested that the isolated microorganism could limit the growth of other bacteria due to competition for nutrients [15]. Building on Tissier’s observations of infants, Élie Metchnikoff, in his work titled “The Prolongation of Life: Optimistic Studies,” notes that diet can alter the intestinal flora, suggesting that a diet rich in fermented foods could combat “intestinal putrefaction.” Metchnikoff emphasized that the effect is not entirely attributable to lactic acid administered on its own, but rather to lactic acid bacteria, which were able to adapt easily to the digestive tract and reduce the number of harmful microbes. Metchnikoff also argued that the consumption of traditionally fermented milk, particularly “Bulgarian yogurt,” based on Grigoroff’s observations in Bulgaria, could be an important source of lactic acid bacteria capable of combating “intestinal putrefaction.” In 1905, Stamen Grigoroff, together with Professor Léon Massol, isolated from Bulgarian yogurt the microbe known as the “Bulgarian bacillus,” later reclassified as *Lactobacillus delbrueckii* subsp. *bulgaricus* [17,18,19]. Another major discovery is attributed to Alfred Nissle, who, during World War I, isolated a nonpathogenic strain of *Escherichia coli* from the feces of a German soldier. Nissle hypothesized that the *Escherichia coli* strain, which later became known as *Escherichia coli* Nissle 1917, exhibited strong antagonistic activity against pathogenic enterobacteria, a fact that could have explained the absence of clinical symptoms in that soldier, unlike his comrades, in the context of exposure to *Shigella* [20].

The earliest use of the term “probiotic,” which has Greco-Latin origins and means “for life,” is attributed to Werner Kollath, who in 1953 described the term “probiotic” in a broader sense, defining it as: organic and inorganic supplements necessary for restoring the health of patients suffering from a form of malnutrition caused by excessive consumption of highly refined foods [21]. In 1965, the term was adapted to a narrower meaning based on Lilly and Stillwell’s in vitro observations on protozoa, when they used it to refer to “growth-promoting factors produced by microorganisms,” capable of prolonging the multiplication phase of other protozoa in culture [22]. In 1974, Parker brought the term closer to its current meaning by describing probiotics as “organisms and substances that contribute to the balance of the intestinal microbiota.” On the other hand, Fuller considered the definition unclear, since the term “substances” could also refer to antibiotics. Thus, he proposed another definition, namely that a probiotic is: “a dietary supplement containing live microorganisms that has beneficial effects on the host animal by improving its intestinal microbial balance” [23]. Three years later, Havenaar and Huis in ’t Veld [24] expanded on this definition, stating that a probiotic is: “a viable, single- or mixed-strain culture of microorganisms that, when administered to animals or humans, exerts a beneficial effect on the host by improving the properties of the endogenous microflora.”

Against the backdrop of the definition’s ongoing evolution, in 2001 the joint FAO/WHO expert consultation formulated a definition that remains a benchmark to this day, describing a probiotic as: “Live microorganisms that, when administered in adequate amounts, confer a health benefit on the host” [25]. However, in 2014, the International Scientific Association for Probiotics and Prebiotics (ISAPP) revisited the definition established by FAO/WHO, clarifying that the term “probiotic” applies strictly to live microorganisms that, when administered in adequate amounts, confer a demonstrated health benefit to the host [26].

In aquaculture, the definition of a probiotic needed to be refined, since the aquatic host is in constant interaction with the microbiota in the rearing environment. In this regard, Verschuere et al. [27] proposed a definition better suited to aquaculture, in which a probiotic was defined as “a live microbial additive that has a beneficial effect on the host by modifying the host-associated microbial community or that of the surrounding environment, by ensuring more efficient feed utilization or by enhancing its nutritional value, by improving the host’s response to disease, or by improving the quality of the surrounding environment.” Gatesoupe [28] attributes the origin of probiotic use in aquaculture to a study conducted by Kozasa in 1986, in which *Bacillus toyoi* spores were tested as a feed additive in aquatic animals. Beneficial effects were reported in Japanese eels infected with *Edwardsiella* species, while increased growth was observed in yellowtail, marking the beginning of probiotic use in aquaculture.

### 3.2. Sources of Probiotics

Microorganisms with probiotic potential can be isolated from a wide range of environmental sources, including freshwater or seawater, bottom sediments, sludge, and soil [29,30,31]. In addition to these external sources, microorganisms with probiotic potential can also be isolated from the gastrointestinal tract and skin mucus of fish. Such host-associated isolates are often considered particularly valuable because they may persist more effectively in the intestine, thereby exerting more durable effects on the host [32,33,34,35]. The search for novel strains has also extended to extreme environments, such as polar habitats. Two strains isolated from Antarctic rock, *Arthrobacter endolithicus* sp. nov. and *Arthrobacter antibioticus* sp. nov., displayed inhibitory activity against several pathogens of interest in fish farming, including *Aeromonas hydrophila*, *Aeromonas sobria*, and *Vibrio anguillarum* [36]. Another strain, designated CLB_ANTARCTIC_008, was isolated from the Antarctic toothfish (*Dissostichus mawsoni*) and exhibited antimicrobial activity against *Aeromonas veronii* TOXIC001 and *Aeromonas hydrophila* [37]. Hypersaline environments may represent another source of microorganisms with probiotic potential [38].

Table 1 summarizes microorganisms with probiotic potential isolated from environmental or host-associated sources, together with their reported effects.

### 3.3. Selection Criteria and Safety Evaluation of Probiotics

**Figure 1:**
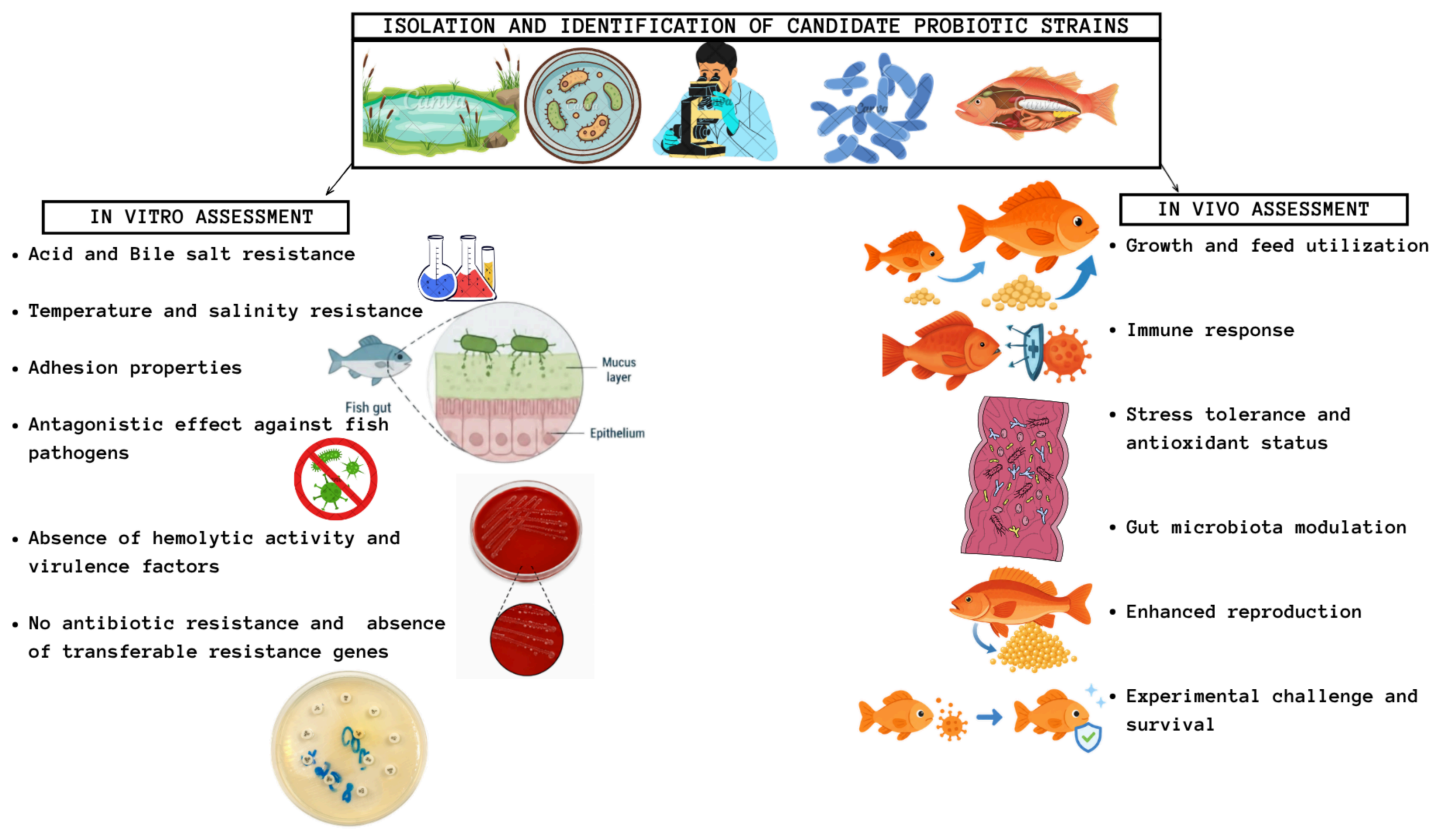
Schematic representation of the main methods for evaluating the safety and efficacy of a microorganism with probiotic potential.

The selection and evaluation criteria for probiotics have been widely discussed by numerous authors [30,55,56,57,58,59,60,61], who emphasize that, in order for a probiotic strain to be safe, it must be tested both in vitro and in vivo by means of various biochemical, immunological, or biomolecular methods. Characterization and selection must therefore be carried out stepwise, from the isolation of the microorganism to the determination of the beneficial effect it can confer on a host, as well as the absence of antibiotic resistance genes, virulence factors, and other potentially harmful traits. In brief, these criteria are: the ability of a probiotic to withstand acidic pH, bile salts, and the host’s body temperature; adhesion, colonization, and autoaggregation capacity; antagonism towards pathogenic/opportunistic agents; the production of digestive enzymes and beneficial metabolites; as well as the absence of hemolytic activity and susceptibility to antibiotics. Figure 1 briefly presents the main methods by which the safety and efficacy of a microorganism with probiotic potential can be evaluated.

An analysis of European Union legislation shows that the use of microorganisms with probiotic potential is subject to a strict regulatory framework, as they cannot be marketed or administered to animals without an authorization and clear evidence of their safety. Regulations No. 1831/2003 [62] and No. 429/2008 [63] state that the safety of administering the additive must be demonstrated for the target species, as well as for the environment and for humans. It is necessary to demonstrate the absence of toxins and virulence factors, along with the absence of transferable antibiotic resistance genes, aspects that must be established through the genetic and phenotypic characterization of the microbial strain. To date, only one commercial product has been approved at European Union level, namely Bactocell^®^ for farmed fish, which contains the strain *Pediococcus acidilactici* CNCM I-4622 [64].

### 3.4. Mechanisms of Action of Probiotics

Probiotics exert their beneficial effects through multiple mechanisms involving both direct interactions with pathogens and effects on host physiology. These mechanisms include modulation of the gut microbiota, competitive exclusion of pathogens, production of antimicrobial compounds, stimulation of the immune response, improved nutrient utilization, as well as interference with bacterial communication systems (quorum sensing and quenching) [65,66]. However, the level of evidence supporting each mechanism varies among studies: some have been demonstrated through direct functional tests, whereas others are supported by associated changes in the microbiota, immune parameters, gene expression, or other physiological responses.

#### 3.4.1. Synthesis of Inhibitory and Antimicrobial Substances

The ability to synthesize inhibitory substances with a bactericidal or bacteriostatic effect represents an important characteristic of strains with probiotic potential and one of the direct mechanisms through which they may inhibit pathogenic microorganisms. Certain probiotic strains are capable of synthesizing a variety of metabolites with bactericidal or bacteriostatic effects, among which siderophores, hydrogen peroxide (H_2_O_2_), lysozymes, proteases and, in particular, bacteriocins (a few examples include enterocin, nisin, plantaricin, and lactacin) may be mentioned [67,68,69,70,71,72,73,74,75,76,77,78]. Other antimicrobial compounds studied in the context of aquaculture include lipopeptides such as surfactin, fengycin, and iturin, as well as reuterin [79,80,81,82]. Indole and its derivatives have also been investigated for their ability to reduce the virulence of bacteria of the genus *Vibrio* [83]. Other strains are able to synthesize lactic acid as well as short-chain fatty acids such as butyric, propionic, and acetic acid, among many others, which can contribute to changes in the intestinal environment and may consequently influence the growth of opportunistic and pathogenic bacteria [75,84,85,86,87,88].

One example of such a metabolite is surfactin, a cyclic lipopeptide synthesized by certain *Bacillus* strains which, in addition to its antimicrobial capacity, also exhibits the ability to inhibit the replication of certain enveloped viruses by interfering with their membrane fusion [89]. Han et al. [90] showed that surfactin inhibited in vitro the cytopathic effect produced by viral hemorrhagic septicemia virus (VHSV) in intestinal epithelial cells of olive flounder (*Paralichthys olivaceus*) at a concentration of 0.3 mg/mL. In a separate in vivo experiment, oral administration of *Bacillus subtilis* prevented detectable cytopathic effects in the intestine, kidney, and spleen [90]. Another example of a microbial metabolite with antiviral activity is prodigiosin, produced by the strain *Serratia marcescens* MS01 isolated from the intestine of largemouth bass (*Micropterus salmoides*), which significantly reduced in vitro the viral titer of Micropterus salmoides rhabdovirus (MSRV) and the expression of viral proteins [91].

Other studies, although less frequent, report the beneficial effect of certain metabolites against fungal and parasitic infections. One example with a major impact in aquaculture is saprolegniosis, in which *Saprolegnia parasitica* is considered the main pathogenic oomycete affecting salmonids [92,93]. In a study carried out by González-Palacios et al. [94], the authors demonstrated that two strains of *Pseudomonas fluorescens*, LE89 and LE141, exerted an inhibitory effect against *Saprolegnia parasitica* infection when administered in the water. Subsequently, in another study by the same authors, it was hypothesized that this inhibitory effect might be associated with the ability of the strains to produce siderophores [95]. In parallel, another study conducted on grass carp (*Ctenopharyngodon idella*) showed that the metabolites of the strain *Pseudomonas monsensis* DBFF02 displayed an inhibitory effect against *Saprolegnia* in vitro [96]. Another example of a metabolite with antifungal activity is lucidin, produced by *Streptomyces zaomyceticus* PQ395286, which inhibited the growth of *Candida albicans* in vitro and reduced the clinical signs associated with infection in African catfish (*Clarias gariepinus*) in vivo [97]. In the case of parasitic infections, the effects associated with probiotics appear to be less well documented, a possible reason being the complex life cycle of many parasitic agents as well as the variability in efficacy among probiotic strains. Overall, the available data show that the efficacy of the surfactant H6 produced by *Pseudomonas* H6 against *Ichthyophthirius multifiliis* has been demonstrated both in vivo and in vitro [98,99]. Experimental observations suggest that one mode of action of the surfactant against *Ichthyophthirius multifiliis* involves interaction with the parasite plasma membrane, accompanied by membrane perforation, possibly resulting in loss of cilia and motility [100]. Antiparasitic activity of the *Pseudomonas* H6 surfactant has also been demonstrated in vitro against the ciliate *Philasterides dicentrarchi* [101] and gill amoebae of the genus *Vannella* [102]. Other examples of metabolites with antiparasitic potential that have shown an inhibitory effect against *Ichthyophthirius multifiliis* include salinomycin, natamycin, and nystatin, produced by different species of the genus *Streptomyces* [103,104,105]. These compounds have demonstrated antiparasitic efficacy in vitro and in vivo. However, the mechanism by which each one acts has not been fully elucidated. Specifically, in the case of natamycin, damaging effects on the tomont membrane have been observed, whereas for salinomycin and nystatin, the mechanisms remain largely hypothetical [103,104,105]. Considering these findings together, the production of various inhibitory substances can be considered an important mechanism of probiotic action. However, the limited evidence regarding the effects of specific compounds against fungal and parasitic infections remains a challenge for the control of these diseases, particularly as these effects have been less extensively investigated. In addition, the mechanisms underlying the antiviral activity of probiotics remain incompletely understood.

#### 3.4.2. Nutrient Competition

Once it reaches the intestine, a microorganism will begin to use nutrient sources that are most often limited, thus entering into competition with various pathogenic or opportunistic agents [106,107,108]. This race for resources can frequently confer an advantage on probiotic microorganisms, since they may be better adapted to that environment and can take up the available nutrients much more rapidly, thereby limiting the growth of pathogenic microorganisms [107]. Among nutrients, the most important one for a microorganism is iron [109]. Iron is involved in many physiological processes, such as energy metabolism, cellular respiration, DNA synthesis, and cell multiplication, and is therefore a valuable resource for bacterial proliferation, yet a limited one within the organism [109,110,111,112]. Given this limited availability of iron in the body, certain microorganisms have developed special mechanisms for its acquisition, in the sense that they produce specific metabolites known as siderophores. Siderophores are metabolites with a high affinity for iron, capable of chelating ferric ions (Fe^3+^) to form soluble complexes that are subsequently taken up by bacteria through specific transport systems. This ability to produce siderophores can provide a competitive advantage in iron-poor environments over microorganisms that lack efficient iron-acquisition systems [113].

A few examples that may be discussed in relation to siderophore production include the study by Lalloo et al. [114], in which *Bacillus cereus* inhibited the growth of *Aeromonas hydrophila* through more efficient iron acquisition. Dobloug et al. [115] likewise observed that the strain *Aliivibrio* sp. Vl2 inhibited the pathogen *Moritella viscosa*, while transcriptomic analysis indicated upregulation of genes related to siderophore production and iron uptake, suggesting a possible role of iron competition. Similarly, the study by Shi et al. [116] shows that *Glutamicibacter* sp. ZY1 inhibited *Vibrio parahaemolyticus* through siderophore-mediated iron competition. Other, earlier studies that addressed the role of competition for and availability of iron are those of Smith and Davey [117], Gatesoupe et al. [118], and Gram et al. [119]. Taken together, the available evidence indicates that iron availability has a major influence on bacterial competition and proliferation, with competition for iron representing an important mechanism through which probiotic microorganisms may limit pathogen growth.

#### 3.4.3. Competition for Colonization Sites and the Influence on Microbiota Balance

Intestinal colonization and modulation of the gut microbiota are frequently described as mechanisms involved in probiotic activity. Indigenous microorganisms have the advantage of being better adapted to the intestinal environment, where they can adhere to the intestinal mucosa. By occupying available niches, they can influence the local microbiome and may limit the growth of opportunistic and pathogenic microorganisms. This interaction can also contribute to the balance of the host intestinal ecosystem [120]. Although it should be noted that this is not a uniform effect, in the sense that sometimes only minor changes are observed, whereas in other cases no significant changes are reported, these results may vary depending on the bacterial strain and possibly the culture system [121]. Following the administration of a probiotic, the literature indicates that changes in the abundance of certain genera may occur in the gut microbiome such as *Bacillus*, *Lactobacillus*, and *Cetobacterium* [59,122].

Several studies have investigated the ability of probiotic strains to colonize the intestine, adhere to intestinal surfaces and influence the intestinal microbiota. Administration of the *Bacillus coagulans* NRS 609 strain to *Cyprinus carpio* demonstrated its ability to colonize the intestine, with the strain persisting for at least 14 days after supplementation was discontinued. The same study also revealed that treatment with the probiotic altered the microbiome by increasing the abundance of the genus *Bacillus* and decreasing that of *Shewanella* [123].

Four probiotic strains (*Enterococcus faecium*, *Pediococcus acidilactici*, *Lactobacillus reuteri*, and *Bacillus subtilis*) were tested individually or in combination in order to assess adhesion to rainbow trout epithelial cells, and *L. reuteri* showed the highest value for inhibition of pathogen adhesion, as demonstrated in vitro by Pillinger et al. [124]. This indicates that the inhibitory effect may depend largely on the strain, since *B. subtilis* exhibited an almost negligible effect compared with *L. reuteri*. Another case in which the strains induced different changes is illustrated by the study by Zhao et al. [125]. The authors note that *Bacillus velezensis* MVCR2 increased the abundance of taxa such as *Ruminococcus*, *Lachnospiraceae* UCG-004, and *Anaerococcus*, while *Lactobacillus sakei* rMA-2 increased the abundance of other taxa such as *Paenibacillaceae* and *Eubacterium hallii*. Ge et al. [126] showed that, in vitro, *Bacillus tequilensis* Bt-CO exhibited the ability to self-aggregate and adhere to intestinal mucus, while in vivo it persisted in the host’s intestine even after administration was discontinued. Another important finding in the same study was the difference in effects between doses: at the 1 × 10^8^ dose, the authors note that the most pronounced beneficial effect on the gut microbiota was observed, whereas at the maximum dose of 1 × 10^9^, the effects were not as clearly evident [126].

From a practical and economic standpoint, the persistence of certain probiotic strains in the fish intestine even after administration has ceased could be an advantage for their use in aquaculture. However, the varying responses observed depending on the strains and doses suggest that colonization capacity and effects on the gut microbiota can vary considerably and, therefore, must be evaluated individually for each probiotic.

#### 3.4.4. The Role of Probiotics in Immune Modulation

Through their administration, probiotics can stimulate and modify certain components of adaptive and innate immunity. A few examples that may be listed are the stimulation of phagocytes, neutrophils, and NK cells. They have the capacity to stimulate the proliferation of B and T lymphocytes and the production of immunoglobulins. They can also modify parameters that are monitored for the evaluation of the immune response, such as lysozyme and complement activity, respiratory burst, and phagocytosis [127,128]. However, based on most studies, these effects are generally presented in broad terms, since they most often depend on the probiotic dose, the duration of the trial, and other factors that can frequently influence the results.

An essential role in the modulation of these mechanisms is played by cytokines, which ensure communication between the components of the immune system and the response to inflammation and infection in fish. Interleukins, interferons, chemokines, and tumor necrosis factors regulate and modulate the immune response when inflammation is present. Following the administration of probiotics in fish, their influence has been observed on pro-inflammatory cytokines such as IL-1β, TNF-α, and IL-6, as well as on anti-inflammatory ones such as IL-10 and TGF-β, supporting their role in the modulation of the immune response. At the same time, these changes alone do not clarify the specific pathways involved [129,130].

Fish are animals that interact constantly with microorganisms from the aquatic environment, and the gills, skin, and gastrointestinal tract represent the first barriers against opportunistic agents. The mucus layer contains antimicrobial peptides, immunoglobulins, and other bioactive molecules that make an active contribution at mucosal surfaces. It can therefore be noted that mucosal immunity has two main roles: first, to combat pathogenic or opportunistic agents, and second, to maintain host homeostasis [131,132]. Mucosal immunity is organized into several categories of mucosa-associated lymphoid tissue (MALT). In teleosts, six subsystems have been described, namely: GALT (gut-associated lymphoid tissue), SALT (skin-associated lymphoid tissue), GIALT (gill-associated lymphoid tissue), NALT (nasopharynx-associated lymphoid tissue), BALT (buccal-associated lymphoid tissue), and PALT (pharyngeal-associated lymphoid tissue). In their analysis, immunoglobulins M, D, T/Z were identified, each with a different distribution and different roles: IgM contributes to both mucosal and systemic immunity; IgT/IgZ are predominantly specialized in mucosal immunity in the species in which they occur. In the case of IgD, the available data are less well established. IgD has been detected in mucosal secretions and may interact with a smaller proportion of the local microbiota, although its specific contribution to mucosal homeostasis remains unclear [133,134,135].

Lactic acid bacteria and species of the genus *Bacillus* are among the most widely used probiotics in aquaculture, together with the yeast *Saccharomyces cerevisiae*. In recent years, however, interest has also been shown in other strains belonging to genera such as *Pseudomonas*, *Pseudoalteromonas*, *Shewanella*, *Aeromonas*, *Vibrio*, *Paenibacillus*, *Streptomyces*, and *Bifidobacterium*. In addition to these, interest has also grown in yeasts such as *Debaryomyces*, *Phaffia*, and *Rhodosporidium* [136,137,138]. In summary, the immunological effects of probiotics have been observed in numerous studies across several fish species. Thus, with regard to the influence of probiotics on mucosal immunity, studies show that the activities of lysozyme, peroxidase, proteases, and alkaline phosphatase can increase, with responses varying according to the strain and dose used, while in other cases even myeloperoxidase activity can increase [139,140,141,142,143]. Other studies show how certain humoral components have been influenced, although the responses differ according to the probiotic and tissue examined. Increased total immunoglobulin levels and agglutination capacity have been reported following probiotic administration [144,145]. However, IgM responses were more variable. Vicente-Gil et al. [145] reported increased IgM levels in skin, intestinal mucus and in serum, whereas Giri et al. [146] found an increase only in skin mucus. In addition, it may be noted that mucus secretion, along with bactericidal activity, increased following the administration of strains such as *Leuconostoc mesenteroides* and *Lactococcus lactis* [147]. At the level of the skin mucus, probiotics can influence protein profile, as in the case of the administration of *Bacillus cereus* QSI-1, which increased complement and lysozyme activity [148]. Similarly, the effects varied depending on the strain and tissue: *Bacillus cereus* NY5 increased SOD activity in the skin, while *Alcaligenes faecalis* Y311 increased alkaline phosphatase activity in the intestine and gills [149]. This strain-dependent variability was also highlighted by Ding et al. [150], where *B. velezensis* DM5 and *B. siamensis* B0E14 increased several markers of skin immunity, although both groups had a 100% mortality rate following infection with *V. harveyi*, whereas *E. faecalis* AT5 significantly reduced mortality. The authors interpret this contrast as a possible consequence of excessive immunostimulation, although this possibility was not experimentally confirmed. Along the same lines, Sanahuja et al. identified changes in pathways associated with B- and T-cell regulation and skin barrier function following *Debaryomyces hansenii* administration, while the antibacterial capacity of the skin mucus was increased in co-culture assays [151]. Similarly, both live and heat-killed *B. subtilis* increased *TNF-α* and *lysozyme* expression, while also reducing the intensity of *I. multifiliis* infestation [152].

With regard to the intestinal immune system, based on the available studies it can be concluded that the effects are essentially: improvements in intestinal morphology and an increase in goblet cells [153,154], as well as cytokine expression, although this can vary between studies. Jang et al. [155] reported an increase in the expression of *TNF-α*, *IL-1β*, and *IL-6* following administration of *Bacillus sp*. SJ-10, while Lei et al. [156] and Hou et al. [157] observed a decrease in *IL-1β* expression, along with an increase in *IL-10* and *TGF-β*.

It is also interesting that several studies mention the influence of probiotics on genes involved in the recognition and activation of the immune response, where increased expression of *TLR3* and *TLR2* was associated by the respective authors with TLR signaling, while changes in MHC II and IgT formed the basis for the hypothesis of an MHC II–CD4–IgT response [158,159,160]. Thus, it can be noted that these studies support the involvement of TLR pathways and the adaptive immune response in probiotic-induced immunomodulation. However, the link between these pathways and the protective effect has not been directly demonstrated, as the mechanisms were inferred primarily from changes in gene expression.

Systemic immune responses have also been reported, including changes in serum IgM, lysozyme and complement activity, respiratory burst, and phagocytic activity [161,162]. Although immunomodulation is recognized as a mechanism of probiotic action in fish, the specific pathways proposed to explain these effects are not yet well understood. Further studies are needed to clarify how these immune responses are related to disease resistance and how probiotics can influence the underlying pathways.

#### 3.4.5. Disruption of Bacterial Cell-to-Cell Communication: Quorum Sensing and Quorum Quenching

Quorum sensing (QS) is a mechanism of intercellular chemical communication through which bacteria coordinate their collective behavior in a manner dependent on cell population density [163]. Bacterial QS systems are usually made up of signaling molecules, the components involved in their synthesis, and receptor proteins through which the regulation of gene expression takes place [164]. The phenomenon is based on the ability of bacteria to produce and release small chemical signaling molecules, known as autoinducers, which increase along with the bacterial population. When a critical threshold is reached, bacteria detect the presence of the autoinducers and provide a coordinated response at the collective level [165]. The first autoinduction system was described in 1970 in the bioluminescent marine bacterium *Photobacterium fischeri*, subsequently reclassified as *Aliivibrio fischeri* [166,167], while in 1994 the term quorum sensing was introduced by Fuqua et al. [168].

To date, depending on the bacterial type, QS communication may be mediated by different signal molecules: Gram-positive bacteria commonly use autoinducing peptides (AIPs), whereas Gram-negative bacteria predominantly use N-acyl homoserine lactones (AHLs). Autoinducer-2 (AI-2) is involved in QS systems of both groups [169,170]. Through QS, bacteria can coordinate a series of collective behaviors, including biofilm formation, activation of virulence-associated mechanisms, coordinated movement across surfaces, production of bioluminescence, secretion of extracellular enzymes, and iron acquisition through the production of siderophores [171,172,173].

Quorum quenching (QQ) can be defined as a mechanism for jamming the various bacterial communication signals coordinated through quorum sensing [173,174]. This interference may act through the enzymatic degradation of the signal molecules or through certain bacterial compounds capable of inhibiting communication [174,175,176]. Given that QS is involved both in virulence and in biofilm formation, QQ can be regarded as a favorable trait of strains with probiotic potential and has been proposed as an alternative strategy that may reduce reliance on antibiotics and the selective pressure for antimicrobial resistance [173,174,177,178,179,180,181,182]. A few examples of strains shown to degrade AHLs and to reduce pathogen abundance, virulence, or infection include: *Bacillus* sp. QSI-1 [183], *Bacillus firmus* sw40 [184], *Bacillus licheniformis* T-1 [185], and *Lactobacillus plantarum* QQ8, *L. casei* QQ10, *Enterococcus faecium* QQ12, and *Bacillus thuringiensis* QQ17 administered in combination [186].

There are also studies that have focused on identifying new strains with QQ traits from the environment for use in aquaculture. Rwezawula et al. [187] report having isolated from pond sediment several bacteria with QQ activity, of which only six were considered to have probiotic potential, including two newly identified strains: *Kocuria crassamentum* sp. nov. KSNUG and *Heyndrickxia crassamentum* sp. nov. HSNUG, both with QQ activity. Similarly, Chen et al. [188] isolated from pond sediment and the intestine of fish, several *Streptomyces* strains, of which approximately 90% carried genes encoding AHL-acylases and nine displayed QQ activity.

### 3.5. Benefits of Probiotic Administration in Fish

#### 3.5.1. Effects on Growth Performance, Gut Microbiome Modulation, and the Production of Beneficial Substances

Probiotics can influence the growth rate of the host by stimulating nutrient absorption as well as by modifying the local microbial population; accordingly, the most commonly studied genera are *Lactobacillus* and *Bacillus* [189,190]. Most studies report that, following the administration of a probiotic to various fish species, positive growth results were recorded. For example, the administration of *Lactiplantibacillus plantarum* promoted growth in several fish species such as common clownfish (*Amphiprion ocellaris*) [191], black sharkminnow (*Labeo chrysophekadion*) [192], common carp (*Cyprinus carpio*) [193], mandarin fish (*Siniperca chuatsi*) [194], largemouth bass (*Micropterus salmoides*) [195], olive flounder (*Paralichthys olivaceus*) [196], Nile tilapia (*Oreochromis niloticus*) [197], and Siberian sturgeon (*Acipenser baerii*) [198]. Another example is *Lacticaseibacillus rhamnosus*, which likewise promoted growth in Nile tilapia (*Oreochromis niloticus*) [199] and in common carp (*Cyprinus carpio*) [200]. Even in combinations of *Lactobacillus acidophilus* with *Saccharomyces boulardii* in Siberian sturgeon (*Acipenser baerii*) [201] and *Lacticaseibacillus casei* with *Saccharomyces cerevisiae* in Asian seabass (*Lates calcarifer*) [202], more significant growth was recorded. The combination of *Limosilactobacillus fermentum*, *Lactiplantibacillus plantarum*, *Lacticaseibacillus casei*, and *Bifidobacterium lactis* likewise improved growth in juvenile Asian seabass (*Lates calcarifer*) [203]. The administration of *B. subtilis* also produced favorable effects on growth in several fish species, namely: grass carp (*Ctenopharyngodon idella*) [204,205], rainbow trout (*Oncorhynchus mykiss*) [206], and African catfish (*Clarias gariepinus*) [207]. Administered in combination with other probiotics as well, beneficial effects on growth have likewise been observed. Thus, the administration of the strains *Bacillus licheniformis*, *Bacillus subtilis*, and *Bacillus pumilus* to European perch (*Perca fluviatilis*) resulted in a high growth gain [208]. Another combination of *B. pumilus*, *B. licheniformis*, and *B. subtilis* in European seabass was considered beneficial for growth [209]. Accordingly, most studies report that WG, SGR, and RGR are increased, while FCR is decreased. It should be noted that there are considerable variations among studies regarding probiotic strains, doses, administration periods, and fish species, which limit the direct comparison of the reported growth responses. In addition to the positive effect on growth, probiotic administration has also been associated with changes in the composition of the local microbiome, which in turn may confer a series of benefits on the host. A few examples include studies in which the administration of a probiotic of autochthonous origin led to an increase in genera such as *Bacillus*, *Lactobacillus*, *Cetobacterium*, and *Paenibacillus*, as well as to a reduction in pathogenic or opportunistic taxa such as *Aeromonas*, *Plesiomonas*, *Streptococcus*, *Staphylococcus*, *Shigella*, and *Acinetobacter* [210,211,212,213,214,215]. In general, the influence on the microbiome depends on the strain used as well as on the health status of the host.

Another beneficial trait of probiotics is their capacity to secrete various compounds, such as enzymes with a nutritional, metabolic, or immunological role. Certain probiotics can therefore synthesize enzymes, vitamins, fatty acids, and polyamines [137,216,217]. However, the ability of a microorganism to synthesize exoenzymes such as amylases, lipases, proteases, xylanases, phytases, and chitinases is an important factor for nutrient digestibility as well as for the development of the host [218,219,220,221]. A few examples of probiotic strains associated with increased digestive enzyme activity in fish include *Bacillus rugosus* NM007, which increased intestinal protease, amylase, and lipase activity in tilapia [222]. Similarly, *Cetobacterium somerae* CGMCC No. 28843 increased trypsin, chymotrypsin, lipase, and α-amylase activity in grass carp [88]. Lee et al. [223] describe several autochthonous strains (*Rummeliibacillus stabekisii* PM227, *Microbacterium oxydans* NB29, and *Microbacterium foliorum* BZ24) isolated from olive flounder, which improved amylase, lipase, and protease activity. In addition to enzymes, the available data indicate that there are also strains capable of synthesizing vitamins or short-chain fatty acids. A first example is *Cetobacterium somerae*, which has been shown to produce vitamin B12 as well as acetic acid [224,225,226]. There are also yeasts, such as *Debaryomyces hansenii*, that can provide compounds such as polyamines, namely spermidine and spermine [216,227]. These polyamines have been associated with different effects. Specifically, spermine has the ability to support growth, protein synthesis, and antioxidant capacity in fish [228], while spermidine, on the other hand, can reduce hepatic lipid accumulation, oxidative stress, and inflammation [229].

#### 3.5.2. Effects on Reproductive Performance

Although data concerning the efficacy of probiotics on reproduction are more limited, there is nevertheless some evidence regarding their effect in various fish species, in the sense that probiotics can stimulate gonadal development and fecundity, can improve gamete quality, and can also enhance fertilization, hatching, and the survival of the resulting fry [230]. In females, the main associated effects relate to higher fecundity, better developed eggs, and greater reproductive success, associated with an improved survival rate of the offspring [231,232,233]. In other cases, the administration of probiotics to females has been associated with a reduction in malformations and in yolk sac defects in the fry [234]. Hormonal changes associated with reproductive status may also be present, such as increased blood levels of LH, FSH, estradiol, and progesterone [235]. In males, the effects mainly concern the improvement of the seminal material and, in some cases, an increase in plasma testosterone [236,237,238]. Probiotics have also been suggested to stimulate gonadal maturation through the hypothalamic–pituitary–gonadal axis, as increased expression of genes involved in the kisspeptin/GnRH/GH/GTH system was observed together with advanced gonadal development [239]. Table 2 summarizes several studies on the effects of probiotics on reproductive performance in fish.

Based on the studies summarized in Table 2, probiotics appear to exert diverse positive effects on reproductive performance, which may differ between males and females and vary with the probiotic strain and dose. However, given the limited number of studies, these findings should be interpreted cautiously. Nevertheless, probiotics may represent a promising complementary approach for improving reproductive performance in fish and reducing mortality in fry.

#### 3.5.3. Effects of Probiotics on Immune Status, Physiological Status, and Disease Resistance

The administration of probiotics can influence the health status of fish through the modulation of the immune response, resistance to pathogens, and biochemical as well as hematological changes, the responses varying according to the strain used, the dose, and other factors [30,58,65]. The following section presents, in summary form, several studies grouped by category starting with the immune response followed by resistance to various pathogens and effects on biochemical and blood parameters, together with their associated effects (Table 3, Table 4 and Table 5).

Overall, from Table 3, Table 4 and Table 5, it can be concluded that the studies presented therein did not establish a general dose–response relationship for the various probiotics administered to fish. Some studies reported a favorable effect with the administration of a higher dose [243,258], while in other studies the optimal effect was observed even at intermediate doses or varied depending on the parameter being monitored [292,297]. It is also worth noting that numerous studies analyzed by us [244,249,250,254,255,256,259,260,262,263] evaluated a single dose of various probiotics, which did not allow us to establish a general optimal dose or to clarify the dose–response variation. For the time being, it can be assumed that the efficacy of a probiotic dose depends to a large extent on the strain used and the host to which it is administered.

#### 3.5.4. Effects of Probiotics on Stress and Antioxidant Status

In aquaculture conditions, particularly in intensive systems, fish are exposed to a wide range of factors that can contribute to stress, such as variations in environmental parameters, various toxic agents, overcrowding in tanks, transport, and human handling. These stressors activate certain nervous and neuroendocrine responses that adapt the organism to the level of stress to which it is exposed. However, the more prolonged the exposure and the longer cortisol levels remain above normal limits, the more the organism may suffer in terms of growth, immunity, and resistance to various pathogens [299]. The more persistent the stress within the organism, the more significant the changes it produces in the balance between reactive oxygen species and the capacity of the antioxidant system, resulting in the onset of oxidative stress. In order to defend itself, the fish organism activates enzymes such as SOD, CAT, and GPx, and probiotics can support this system by stimulating antioxidant capacity and limiting reactive oxygen species [300].

The administration of probiotics has been associated in several studies with increases in total protein, albumin, and globulins, and with the stimulation of superoxide dismutase (SOD), catalase (CAT), and glutathione peroxidase (GPx) activity, as well as of total antioxidant capacity (TAC) [301,302,303,304]. Probiotics can also decrease the activity of aspartate aminotransferase (AST) and alanine aminotransferase (ALT), as well as the level of malondialdehyde (MDA) [302,305]. Probiotics are considered to possess several mechanisms through which they help the host to counteract oxidative stress; the mechanisms mentioned are the chelation of metal ions, modulation of the gut microbiota, synthesis of antioxidant metabolites, and reduction in the formation of reactive oxygen species [298]. More recently, certain probiotics have been associated with changes in Nrf2–Keap1-related gene expression, alongside changes in *SOD*, *CAT*, and *GPx* expression [204,306,307], suggesting a possible involvement of this pathway in antioxidant effects.

Table 6 briefly presents several studies in which probiotics attenuated the impact of various physical and chemical stress factors.

The studies in Table 6 briefly outline the beneficial effects of probiotics under various physicochemical stress conditions. However, of particular relevance are those that have sought, using different doses, to counteract the effects of various heavy metals and pesticides present in aquaculture [323]. Thus, some studies used concentrations considered relevant to the aquatic environment [314,316], while others used concentrations selected to produce measurable toxic effects under controlled experimental conditions [312,313,317,319]. Although these studies have shown that different probiotics mitigate the effects of various chemical stressors, it cannot be determined whether they would have the same relevance under uncontrolled conditions, such as on farms with different aquaculture systems. Therefore, future studies should at least attempt to assess the relevance of probiotics in combating chemical factors under commercial aquaculture conditions.

#### 3.5.5. The Role of Probiotics in Improving Water Quality

In addition to the effects presented above, probiotics can also improve certain water parameters through the degradation of organic matter, the processing of nitrogenous compounds, and the control of opportunistic or pathogenic microorganisms [121,324]. Strains of the genus *Bacillus* are therefore frequently used for this purpose, either alone or in combination with *S. cerevisiae*, as well as nitrifying bacteria of the genera *Nitrosomonas* and *Nitrobacter* [324,325]. More recently, certain studies on biofilters in RAS have highlighted the importance of comammox bacteria of the genus *Nitrospira*, which are capable of complete ammonia oxidation and have been reported as dominant members of nitrifying communities in some systems [326,327,328].

Studies show that probiotics can influence water quality by reducing nitrogenous compounds, phosphorus, the organic load, chemical and biochemical oxygen demand (COD/BOD), hydrogen sulfide (H_2_S), and pathogenic microorganisms. Probiotic administration has also been associated with changes in pH and dissolved oxygen, although these responses vary among studies [59,302,329]. Table 7 below briefly presents several studies on the effects of different probiotics on water quality in aquaculture systems.

Ammonia reduction was the most consistent effect observed across the analyzed studies in Table 7, while nitrite and nitrate showed more variable responses. Thus, it can be suggested that probiotic efficiency for water quality depends strongly on the microorganisms used and may support the use of multistrain combinations to obtain a stronger effect.

## 4. Prebiotics

### 4.1. Definition and Historical Evolution

The term “prebiotic” was first introduced in 1995 by Gibson and Roberfroid [340], who formulated an initial definition, namely that “a prebiotic is a non-digestible food ingredient that beneficially affects the host by selectively stimulating the growth and/or activity of one or a limited number of bacteria in the colon, and thus improves host health.” Subsequently, in 2004, the initial definition was refined, stating that a prebiotic is a selectively fermented ingredient that allows specific changes, both in the composition and/or activity of the gastrointestinal microflora, that confers benefits upon host wellbeing and health [340]. At the same time, Gibson et al. [341] emphasized that the classification of a food compound as a prebiotic must meet three essential criteria: (a) it must resist gastric acidity, hydrolysis by mammalian enzymes, and gastrointestinal absorption; (b) it is fermented by the intestinal microflora; and (c) it selectively stimulates the growth and/or activity of intestinal bacteria associated with health and wellbeing. Roberfroid [342] emphasized that not all non-digestible carbohydrates can be considered prebiotics according to the established selection criteria, and that at that time only inulin and trans-galacto-oligosaccharides (TOS) fulfilled the conditions then in force. However, in 2008, the Food and Agriculture Organization (FAO) considered the initial definitions of the prebiotic to be far too restrictive and suggested a different approach to the definition, according to which “a prebiotic is a non-viable food component that confers a health benefit on the host associated with modulation of the microbiota” [343]. In 2010, Roberfroid et al. [344] did not create an entirely new definition for prebiotics, but rather sought to reinforce and broaden the existing concept, highlighting that the basis of the prebiotic effect lies in the selective stimulation of the growth and/or activity of a restricted group of microorganisms within the intestinal microbiota, correlating this with significant advantages for host health. In 2017, the International Scientific Association for Probiotics and Prebiotics (ISAPP) sought to provide greater clarity regarding the initial definition. The new definition aimed to preserve the essence of the earlier one while adapting it to more recent information concerning the microbiota, the diversity of the microorganisms involved, and the effects that may also occur outside the gastrointestinal tract. In other words, the prebiotic was characterized as “a substrate that is selectively utilised by host microorganisms conferring a health benefit” [345]. In conclusion, this definition placed emphasis on the selective utilization by host microorganisms and on the presence of a demonstrable health benefit, while broadening the concept to include other types of substances and various microbial sites, not only conventional carbohydrates and the intestine.

In aquaculture, the first reports on the testing of functional additives are represented by research from the 1990s that followed two distinct paths. Several studies focused on the direct stimulation of immune responses using yeast-derived glucans and other compounds, including chitosan [346,347,348]. In 1995, however, Kihara et al. [349] demonstrated that lactosucrose was able to exert a trophic effect on the intestine in vivo and that, in vitro, it could also be fermented by the gut microbiota of red seabream. This may be regarded as a precursor to the application of the prebiotic concept in aquaculture.

### 4.2. Common and Emerging Types of Prebiotics Used in Aquaculture

At present in aquaculture, among the best known and most extensively investigated prebiotics that have shown positive effects on the gut microbiota and on host health are: inulin, fructo-oligosaccharides (FOS) together with short-chain fructo-oligosaccharides (scFOS), mannan-oligosaccharides (MOS), and β-glucans (BG), the latter for their immunostimulatory role [350,351]. Other oligosaccharides include galacto-oligosaccharides (GOS), xylo-oligosaccharides (XOS), arabinoxylan-oligosaccharides (AXOS), and isomalto-oligosaccharides (IMO). Among these, interest in XOS has grown in recent years, relative to the still limited number of available studies [352,353].

In recent years, interest has also increased in natural polysaccharides and their derivatives, such as chitin, chitosan, and chito-oligosaccharides [354,355], fucoidan [356], and laminarin [357]. In addition, research also points to the emergence of alginate oligosaccharide [358,359], bacterial exopolysaccharides [354,360], and pectic oligosaccharides (POS) [354]. To provide a synthesis of these compounds, the main categories of known and emerging prebiotics used in aquaculture are presented in Table 8.

### 4.3. Safety and Regulatory Considerations of Prebiotics

Unlike probiotics, prebiotics are not classified as a distinct category under European Union legislation, with the result that their regulatory status varies depending on their use and marketing. When marketed as feed additives, they are subject to Regulations (EC) No 1831/2003 [62] and (EC) No 429/2008 [63], under which safety must be demonstrated for the target animal and the environment, and evidence of a beneficial effect must also be provided. However, if they are classified as feed materials, in accordance with Regulation (EC) No 767/2009 [371], they must be safe, suitable for their intended use and correctly labeled. It is also important that claims regarding the nutritional characteristics and functions of the product are objective, verifiable and scientifically substantiated [371]. Regulation (EU) No 68/2013 [372] also establishes the Catalogue of Feed Materials, which lists compounds used as prebiotics such as inulin, fructo-oligosaccharides (FOS) and xylo-oligosaccharides (XOS).

### 4.4. Mechanisms of Action of Prebiotics

**Figure 2:**
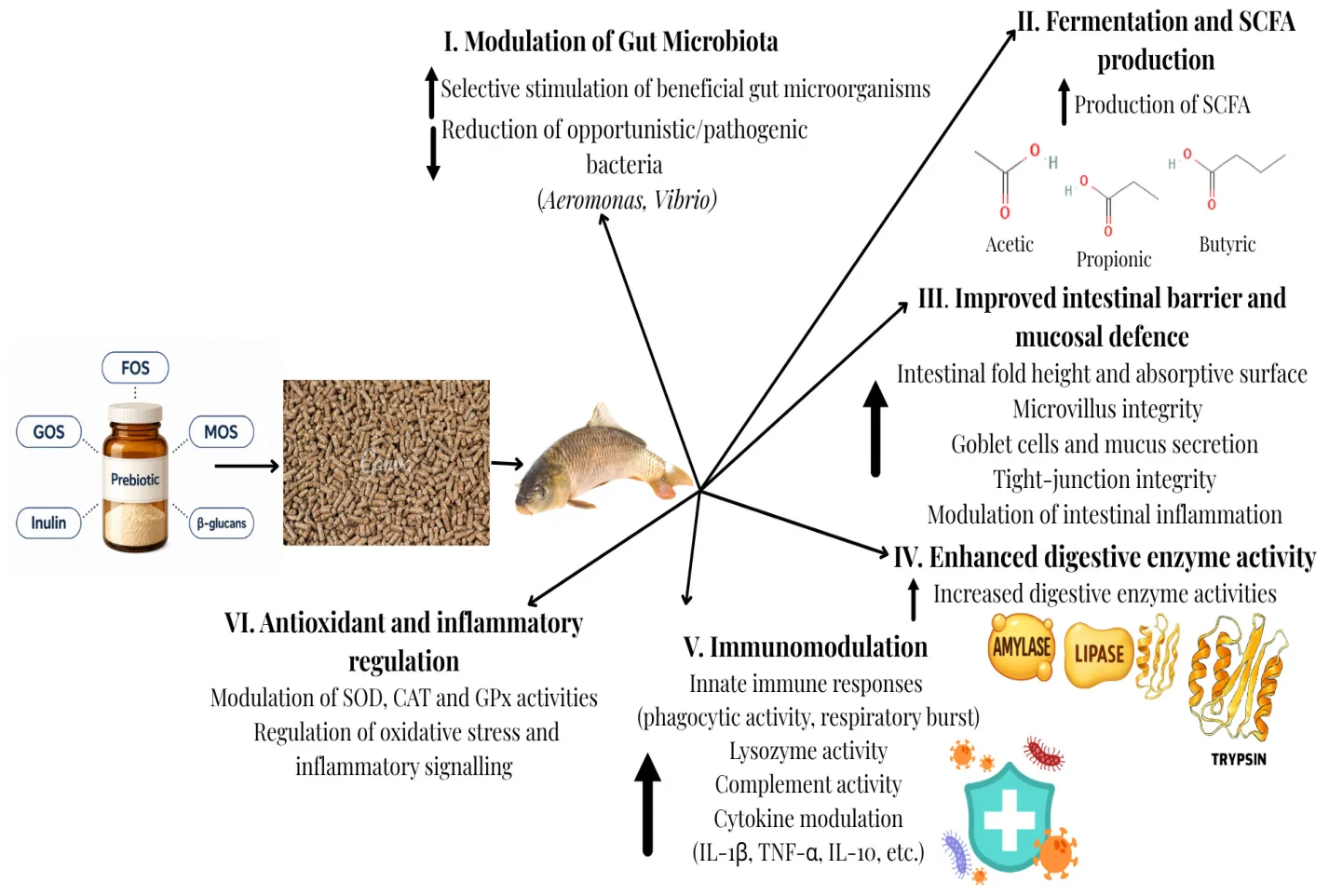
Main proposed mechanisms of action of prebiotics in fish.

Prebiotics act mainly in the gut, where their fermentation can produce metabolites such as short-chain fatty acids, which are associated with changes in the gut microbiota, intestinal barrier, and immune response [373]. Prebiotics can also influence digestive enzyme activity and the antioxidant system, although responses vary with the compound, dose, and fish species [374]. The main proposed mechanisms are presented in Figure 2.

### 4.5. Effects of Prebiotic Administration in Fish

#### 4.5.1. Effects on Growth, Gut Microbiota, and Intestinal Function

Prebiotics can support fish growth and serve as a substrate for the development of beneficial microorganisms in the intestine, although their effects depend on the compound used, the dose, and the fish species [375]. One example is the administration of XOS to hybrid tilapia [376] and to rainbow trout [377], which improved growth and enzyme synthesis and stimulated the proliferation of the genera *Lactobacillus* and *Bacillus.* Table 9 presents a selection of studies that have reported such beneficial effects in various farmed fish species.

Based on the studies summarized in Table 9, we observed that different prebiotics improved both growth and intestinal health, but dose was a key factor in the response. In most cases, intermediate doses produced the best results, while the highest doses provided no additional benefit [382,383,385] or resulted in a weaker response [378,381,384].

#### 4.5.2. Effects of Prebiotics on Immunity and Resistance to Pathogens

In addition to their other effects, prebiotics have been associated with changes in innate immune parameters and increased resistance to different pathogens. These findings have encouraged their evaluation as part of strategies aimed at reducing antibiotic use in aquaculture [352]. β-glucans and mannan-oligosaccharides (MOS) are among the prebiotics investigated for their effects on immune responses and disease resistance in fish [128,386]. Table 10 summarizes several studies reporting the immunomodulatory effect and the increase in resistance to pathogens in farmed fish.

As far as can be observed in Table 10, prebiotics may increase resistance to different pathogens, although the response varies with the prebiotic used, the dose, and the duration of administration. Higher doses did not consistently provide greater protection, and in several studies, lower or intermediate levels produced comparable or even better outcomes [387,389,394,395].

#### 4.5.3. Effects of Prebiotics on Antioxidant Status and Stress Resistance in Fish

Prebiotics can help fish maintain their oxidative balance by stimulating enzymes such as SOD, CAT, and GSH-Px, while also reducing lipid peroxidation. In other words, a stronger antioxidant response may contribute to better tolerance to stress factors frequently encountered in intensive rearing systems, such as accumulated ammonia or crowding in tanks. The effect is not uniform, however, and may depend on the type of prebiotic used, dose, duration of administration, and rearing conditions [397]. The application of prebiotics has thus been associated with a reduction in the stress caused by various stressors, based on the synthesis of the studies presented in Table 11.

Prebiotics, like probiotics, may influence the effects of certain heavy metals and pesticides. As we discussed in the case of probiotics, the available studies [403,405,406,407] suggest that prebiotics may help mitigate the effects of these chemical agents. However, the data are still limited and come mainly from controlled experimental conditions, so we cannot say with certainty that the same effects would hold true under farm conditions until this is confirmed by further studies.

## 5. Conclusions

Both probiotics and prebiotics are, to date, valuable additives, given the wide range of effects they can confer on the host and on the environment. However, these effects depend on the type used, the dose, the duration of administration, and the species to which they are administered, with responses ranging from favorable outcomes to no significant changes.

Future research should focus on testing multi-strain probiotics, in which each strain provides a well-defined effect, and should also take into account the compatibility between the selected strains. In addition, combinations of probiotics with prebiotics, that is, synbiotics, should be studied far more intensively, given the need for compatibility between the two components.

Probiotics and prebiotics may therefore represent promising complementary strategies that could help reduce reliance on antibiotics in aquaculture, although, as mentioned above, the optimal dose and duration of administration still need to be established.

## Figures and Tables

**Table 1:** Microorganisms with probiotic potential: isolation source and associated effects.

Probiotic Candidate	Source of Isolation	Target Species	Host Effects	Reference
*Pediococcus* *pentosaceus* SL001	Soil	Grass carp (*Ctenopharyngodon idella)*	Improved growth (WGR: 15.06% vs. 11.69% C); enhanced resistance to *Aeromonas hydrophila* infection (survival: 48.3% vs. 10% C; modulation of the gut microbiota through proliferation of the genus *Pediococcus* and reduction in pathogenic genera	[39]
*Streptomyces* *chartreusis* KU324443	Soil	Common carp *(Cyprinus carpio)* (juveniles)	Improved growth (WG: 103.57–135.25% vs. 83.53% C, ↓ FCR (1.78–2.12 vs. 2.39 C); serum and mucosal immunity were stimulated at all tested doses, and the expression of immune and antioxidant genes increased at a dose of 10^7^ CFU/g	[40]
*Bacillus velezensis* MT9	Soil	Nile tilapia *(Oreochromis niloticus)*	Favorable modulation of the gut microbiota through a decrease in bacteria of the genera *Aeromonas*, *Vibrio*, and *Escherichia*/*Shigella* and proliferation of those belonging to the genus *Romboutsia* (25.63% vs. 7.20% in C at 90 days)	[41]
*Paenibacillus ehimensis* NPUST1	Water	Nile tilapia *(Oreochromis niloticus)*	Improved growth performance (↑ FW (52.9–55.6 g vs. 37.3 g in C), ↓ FCR (1.08–1.10 vs. 1.38 C), ↑ FE), ↑ survival after *Aeromonas hydrophila* and *Streptococcus iniae* challenge (*S. iniae*: 50–51% vs. 17.8% C), stimulation of non-specific immunity	[42]
*Lactococcus cremoris* *WA2-67* and *WA2-67(pJFQIAI) +* non-bacteriocinogenic *WA2-67 ΔnisZ*	Water	Rainbow trout (*Oncorhynchus mykiss*) + RTgutGC models (intestinal cells) + splenic leukocytes	The bacteriocinogenic strains stimulated the intestinal and splenic immune response. The expression of cytokines and antimicrobial peptides increased. ↑ nitric oxide production (~4–5 μM vs. ~2–2.5 μM in C).	[43]
*Kluyveromyces lactis* M3	Hypersaline sediment	Gilthead seabream *(Sparus aurata)*	↑ mucosal immunity (bactericidal activity of skin mucus against *Vibrio* spp., against *V. parahaemolyticus* (~22–27% vs. ~6% in C after 15 days); no cytotoxic effects on leukocytes were observed	[44]
*Bacillus* *licheniformis*	Mud	Common carp *(Cyprinus carpio)*	↑ growth performance at 10^8^ CFU (FBW 112.80 vs. 76.26 g C; WGR 186.88% vs. 99.73% C; FCR: 1.07 vs. 1.86 C); the gut microbiota was modulated through an increase in *Firmicutes* and a decrease in pathogenic ones; ↑ immune response following *A. hydrophila* challenge	[45]
*Saccharomyces* *cerevisiae*	*S. cerevisiae*-based fermentation product (ESTAQUA^®^)	Channel catfish *(Ictalurus punctatus)*	↑ growth performance (FW: 65.09 vs. 60.92 g in C; (WG: 165.91% vs. 147.95% C), ↓ FCR (1.56 vs. 1.76 C); improved intestinal integrity, with development of goblet cells; ↓ hepatic inflammation; ↓ mortality following *Aeromonas* spp. infection (62.5% vs. 100% in C at 28 h)	[46]
*Lactococcus lactis* LBM15	Intestine	*Largemouth bass (Micropterus* *salmoides)*	Inhibited the growth of several bacterial pathogens, including *Aeromonas hydrophila*, *Vibrio* spp., and *Edwardsiella tarda* (inhibition zone: 18.22 mm against *V. alginolyticus* and 12.35 mm against *A. hydrophila*) Reduced pathogen adhesion to the intestinal mucus, with a marked effect against *A. hydrophila* (inhibition: 76.07%). No mortality or clinical changes were recorded in the safety trials.	[47]
*Bacillus subtilis* (Y2) + *Bacillus amyloliquefaciens* (Y8)	Intestine	Hybrid sturgeon (*Acipenser baerii* × *A. schrenckii*), 8 weeks.	↑ growth performance, ↓ FCR; the treated groups showed morpho-structurally more developed musculature and longer villi; ↓ mortality following *Aeromonas veronii* infection (46.66% in Y8 and 56.66% in LY2 vs. 86.66% in C).	[48]
*Bosea* sp. OX14 + *Flavobacterium* sp. GL7	Skin mucus	Rainbow trout (*Oncorhynchus mykiss*)	Inhibited the growth of *Flavobacterium psychrophilum* in vitro (OX14 reduced *F. psychrophilum* growth by 63–65%, while GL7 reduced it by 47–68% compared with C).	[49]
*Acinetobacter* KU011TH	Skin mucus	Bighead catfish (*Clarias macrocephalus*) (fingerlings 15 g) and (juveniles 150 g)	Simultaneous administration in feed and water led to ↑ growth performance (WG: 25.19 vs. 19.45 g in C), ↓ FCR (2.38 vs. 3.09 C), ↑ immune response and skin mucous cell density; ↑ survival after *Aeromonas hydrophila* challenge (56.7–57.7% vs. 30.0% in C)	[50]
*Lactococcus lactis* (Q-8/Q-9/Z-2)	Intestinal content of common carp (*Cyprinus carpio*)	Common carp (*Cyprinus carpio*—33 g), 8 weeks.	↑ growth performance (WG 220.24–246.49% vs. 203.24% C), ↑ survival (87.78–90.00% vs. 82.22% in C), ↓ *A. hydrophila* adhesion to the intestinal mucus; reduced post-infection inflammatory response, with ↑ IL-10 and TGF-β	[51]
*Lactococcus lactis L19 and Enterococcus faecalis W24*	Intestine	Northern snakehead *(Channa argus)*	↑ growth performance FBW (38.87 vs. 33.67 g in C), WG (308.40% vs. 254.72%), most notably for *L. lactis* L19; ↑ humoral immunity (IgM, lysozyme, complement); ↑ survival after *Aeromonas veronii* challenge (L19: 63.3% vs. 18.3% in C).	[52]
*Bacillus velezensis D-18*	Fish farm wastewater	European seabass *(Dicentrarchus labrax)*	In vitro inhibition of the pathogen *Vibrio anguillarum (30%)*; ↓ pathogen adhesion to the intestinal mucus (60%); ↑ survival following *Vibrio anguillarum* infection (78% vs. 35% in C).	[53]
*Bacillus subtilis BSth-5 and BSth-19*	Sturgeon intestine	Dabry’s sturgeon (*Acipenser dabryanus*)	↑ SR during the feeding period (95.0–95.83% vs. 90.0% in C); ↑ serum antioxidant activity was stimulated (T-AOC, T-SOD) and ↓ MDA; ↑ serum immunity (IgM and lysozyme); ↑ survival following *Aeromonas hydrophila* infection (53.33–66.67% vs. 33.33% in C).	[54]

Note: The arrows ↑ and ↓ indicate increased and decreased, respectively; C: Control.

**Table 2:** Effects of probiotics on reproductive performance in fish.

Fish Species	Probiotic	Dose and Duration of Administration	Main Reproductive Effects	Reference
Rainbow trout (*Oncorhynchus mykiss*)— female broodstock	Bio-Aqua^®^ multi-species probiotic	1 × 10^9^, 2 × 10^9^ or 4 × 10^9^ CFU/kg feed; 8 weeks	4 × 10^9^ CFU/kg feed: ↑ reproductive output (working fecundity: 7976.9 vs. 5446.6 eggs/fish C; egg diameter: 4.9 vs. 4.4 mm C) and improved egg and offspring quality, with ↑ fertilization (98.2% vs. 94.4% C), eyed-egg survival (98.8% vs. 91.0% C), and alevin survival (94.3% vs. 90.6% C); best overall reproductive performance at this dose.	[231]
Silver catfish (*Rhamdia quelen*)—males and females	*Bacillus subtilis* CBMAI 926 + *Bacillus cereus* CBMAI 988	0.15, 0.30, 0.45 or 0.60 g/kg feed; 90 days	Males: at 0.45 g/kg feed, ↑ relative seminal volume (51.17 vs. 31.84 mL/kg C); at 0.60 g/kg, ↑ sperm cell survival (96.50% vs. 93.66% C). Females: ↑ spawning at 0.15, 0.45 and 0.60 g/kg (80–90% vs. 50% C) and ↑ fertilization at 0.30–0.60 g/kg (98.25–98.85% vs. 79.81% C), with stimulated germ-cell development; 0.60 g/kg was recommended for overall reproductive performance.	[232]
African catfish (*Clarias gariepinus*)—females	*Bacillus* sp. NP5	10^6^ or 10^8^ CFU/g feed; 8 weeks	1 × 10^8^ CFU/g feed: accelerated gonadal maturation (GSI: 14.74% vs. 6.03% C at week 4) and ↑ fecundity (243,605 vs. 148,828 eggs/kg C), hatching (80.62% vs. 59.30% C); larval survival (87.31% vs. 71.28% C); rematuration was shortened to 4 vs. 6 weeks C, with 100% mature broodstock;	[233]
Rainbow trout (*Oncorhynchus mykiss*)—female broodstock	Bio-Aqua^®^ multi-species probiotic	1 × 10^9^, 2 × 10^9^ or 4 × 10^9^ CFU/kg feed; 8 weeks	2–4 × 10^9^ CFU/kg feed: ↓ total offspring malformations (0.4–0.3% vs. 0.6% C) and yolk-sac resorption defects (30.2–28.2% vs. 46.1% C), indicating improved offspring quality	[234]
Red tilapia (*Oreochromis niloticus × O. mossambicus*)—males and females	*B. subtilis* + *B. licheniformis* (1:1)	0.01, 0.02 or 0.03 g/m^3^ water; spawning monitored for 20 days	↑ GSI in males (4.95% vs. 3.36% C) and females (5.02% vs. 4.06% C), egg diameter (1.69 vs. 1.17 mm C), fry production (1478.33 vs. 1130 fry/fish C), and fry weight (16.89 vs. 16.05 g C), together with enhanced gonadal maturation and upregulation of reproduction-related genes at the dose of 0.03 g/m^3^	[235]
Goldfish (*Carassius auratus*)—males	*Pediococcus acidilactici* (Bactocell^®^)	0.1, 0.2 or 0.3% of diet; 180 days	At a dose of 0.2–0.3%: ↑ sperm motility (33.13–33.23% vs. 31.23% C) and sperm density (5.83–6.10 vs. 5.50 × 10^9^ cells/mL C) Dose of 0.3%: females showed the highest fecundity (1867 eggs), fertilization (78%), and hatching rate (75.33%), while C females did not spawn, indicating improved gamete quality and reproductive performance.	[240]
Atlantic salmon (*Salmo salar*)—males	*Pediococcus acidilactici* MQ 18/5 (Bactocell^®^)	1 × 10^6^ CFU/g feed; 60 or 120 days	↑ sperm concentration (20.13 vs. 8.76 × 10^9^ sperm/mL C) and embryo viability (64.4–78.1% vs. 36.7–56.5% C), while embryonic mortality decreased (1.4–23.2% vs. 9.4–48.3% C), showing improved reproductive performance and offspring survival, particularly after 120 days	[238]
Silver barb (*Barbonymus gonionotus*)—males and females	*E. xiangfangensis* (GFB-1), *P. stutzeri* (GFB-2), *B. subtilis* (GFB-3), *C. freundii* (GFB-4), and *P. aeruginosa* (GFB-5)	1.06 × 10^9^ CFU/kg feed; 60 days	*B. subtilis* increased GSI (~11.7% vs. ~10.4% C), ova production (~211 vs. ~190 ova/g body weight C), fertilization (~68% vs. ~60% C), and hatching (~58% vs. ~51% C), and resulted in the highest larval survival among the individual strains tested.	[241]
Zebrafish (*Danio rerio*)—females	*Pediococcus acidilactici* Bactocell^®^ CNCM MA 18/5 M	1 × 10^6^, 2 × 10^6^, 4 × 10^6^ or 8 × 10^6^ CFU/g feed; 60 days	At 4 × 10^6^: ↑ GSI (20.07% vs. 8.15% C), absolute fecundity (249 vs. 103 eggs C), working fecundity (215.3 vs. 46.6 eggs C), and hatching (86% vs. 45% C); relative fecundity was highest at 1 × 10^6^ CFU/g feed (420 vs. 277 C).	[242]

Note: C: Control; GSI: gonadosomatic index; ↑: increase; ↓: decrease.

**Table 3:** Effects of probiotics on the immune response in farmed fish.

Fish Species	Probiotic	Dose and Duration of Administration	Main Effects	Reference
Tongue sole (*Cynoglossus semilaevis*)	*Bacillus subtilis*, *B. licheniformis,* *Lactobacillus plantarum*	5 × 10^8^ or 2 × 10^9^ CFU/kg feed, 21 days	At 2 × 10^9^ CFU/kg feed: ↑ lysozyme activity (~7.80 vs. ~7.51 μg/mL C), complement C3 (~0.095 vs. ~0.068 mg/mL C), ALP activity (~7.9 vs. ~2.6 U/L C) and ACP activity (~12.3 vs. ~8.8 U/L C) after 21 days.	[243]
Olive flounder (*Paralichthys olivaceus*)	*Bacillus subtilis* E-TSA-3, *Enterococcus faecium* YPD-N2, *Pediococcus pentosaceus* MRS-A4-1	6.5 × 10^7^ CFU/g feed, 35 days	*B. subtilis* showed higher phagocytic activity (0.066 vs. 0.054 C), while *P. pentosaceus* significantly ↑ phagocytic activity (0.070 vs. 0.054 C); *E. faecium* and *P. pentosaceus* significantly ↑ lysozyme activity (21.52 and 19.13 vs. 4.55 U/mL C)	[244]
Mrigal carp (*Cirrhinus mrigala*)	*Bacillus licheniformis*	1 × 10^6^, 5 × 10^6^, 1 × 10^7^, or 5 × 10^7^ CFU/g feed, 30 days	At 1 × 10^7^ CFU/g feed: ↑ phagocytic ratio (~18 vs. ~7% C), phagocytic index (~1.95 vs. ~1.45 C), respiratory burst activity (~0.30 vs. ~0.24 C), and serum bactericidal activity (~60% vs. ~40% C).	[245]
Hybrid grouper (*Epinephelus fuscoguttatus* ♀ × *E. lanceolatus* ♂)	*Bacillus subtilis* BS, 6-3-1 and HAINUP40	1 × 10^8^ CFU/g feed, 42 days	BS showed an anti-inflammatory/anti-apoptotic gene-expression profile (*tnfα*: ~0.30 vs. ~0.90 C); 6-3-1 showed a weaker anti-inflammatory/anti-apoptotic gene-expression profile (*p65*: ~0.50 vs. ~1.00 C); HAINUP40 showed a pro-inflammatory/pro-apoptotic gene-expression profile (*il-1β*: ~1.50 vs. ~1.00 C).	[246]
Striped catfish (*Pangasianodon hypophthalmus*)	Commercial EPM mixture of probiotic microorganisms	1.5–4.5% of the feed, 90 days	At 4.5% EPM: ↑ phagocytic activity (7.33% vs. 5.16% C), lysozyme activity (502 vs. 412 U/mL C), and oxidative burst/NBT response (~0.49 vs. ~0.09 C).	[247]
Nile tilapia (*Oreochromis niloticus*)	*Lactobacillus reuteri* ATCC 53608	1 × 10^9^–1 × 10^11^ CFU/kg feed, 56 days	At 1 × 10^10^ CFU/kg feed: ↑ serum lysozyme activity (730.6 vs. 322.9 U/mg protein C); ↑ intestinal anti-inflammatory *TGF-β* expression (~1.9 vs. ~1.0 C) and ↓ pro-inflammatory *IL-1β* expression (~0.75 vs. ~1.15 C).	[248]
Yellow catfish (*Pelteobagrus fulvidraco*)	*Bacillus amyloliquefaciens*	1 × 10^6^ CFU/g feed, 4 weeks	At 1 × 10^6^ CFU/g feed: ↑ respiratory burst after 2 weeks (~0.085 vs. ~0.065 C) and lysozyme activity after 4 weeks (~0.68 vs. ~0.29 U/mL C); ↑ intestinal *IgM* (~8.2 vs. ~3.1 C), *CTL* (~9.0 vs. ~3.2 C), and *STAT-1* (~2.0 vs. ~1.1 C) expression after 4 weeks.	[249]
Common carp (*Cyprinus carpio*)	*Lactobacillus plantarum* and *L. rhamnosus*	1 × 10^8^ CFU/g feed, 180 days	After 90 days: ↑ total Ig (~12 vs. ~6 mg/mL C); after 180 days: ↑ respiratory burst/NBT activity (~0.50 vs. ~0.38 C).	[250]
Barramundi (*Lates calcarifer*)	*Bacillus subtilis*— Biota-Pro^®^	5 × 10^9^ CFU/kg feed or 3 × 10^9^ CFU/L water, 60 days	↑ intestinal *MHC I* (~9.0 vs. ~5.5 C), *IL-1β* (~11.3 vs. ~5.8 C), and *IL-10* expression (~8.8 vs. ~6.5 C) after 60 days.	[251]
Grass carp (*Ctenopharyngodon idella*)	*Bacillus velezensis* B8	1 × 10^7^ or 1 × 10^9^ CFU/g feed, 4 weeks	At 1 × 10^9^ CFU/g feed: ↑ serum AKP activity (~100 vs. ~68 U/mL C; 2 weeks), splenic *IgM* expression (~2.2 vs. ~1.0 C; 4 weeks), and renal *TNF-α* expression (~2.7 vs. ~1.2 C; 4 weeks).	[252]
Large yellow croaker (*Larimichthys crocea*)	*Clostridium butyricum*	0.10%, 0.20%, 0.40% in feed (5 × 10^9^ CFU/g); 30 days	At 0.10–0.20%: ↑ LYZ activity (1.32–2.09 vs. 0.60 U/mg protein C) and AKP activity (2091.23–2456.50 vs. 1014.21 U/g protein C); at 0.10%, ↑ ACP activity (1179.07 vs. 663.78 U/g protein C).	[253]
European seabass (*Dicentrarchus labrax*)	*Bacillus velezensis* D-18	1 × 10^6^ CFU/g feed; 30 days	↑ serum bactericidal activity (~86% vs. ~34% C), lysozyme activity (~200 vs. ~112 μg/mL C), and phagocytic activity (~91% vs. ~52% C) after 30 days.	[254]
Rohu (*Labeo rohita*)	*Bacillus methylotrophicus*, *B. amyloliquefaciens* and *B. licheniformis*, individually or in combination	1 × 10^7^ cells/g feed; 60 days	*B. methylotrophicus* ↑ lysozyme activity (17.6 vs. 14.7 μg/mL C) and phagocytic activity (30.90% vs. 24.62% C); *B. amyloliquefaciens* ↑ lysozyme activity (16.3 vs. 14.7 μg/mL C) and phagocytic activity (28.82% vs. 24.62% C); *B. licheniformis* produced the strongest single-strain response, with ↑ lysozyme (19.4 vs. 14.7 μg/mL C), ACP (187.40 vs. 145.23 ACH_50_ units/mL C), and phagocytic activity (35.40% vs. 24.62% C) after 60 days.	[255]
Golden pompano (*Trachinotus ovatus*)	*Bacillus pumilus* A97	1 × 10^8^ CFU/g feed; 56 days	↑ lysozyme activity (~520 vs. ~350 U/mL C), intestinal *TLR8* expression (~4.4 vs. ~1.5 C), and renal *TLR9* expression (~4.2 vs. ~1.0 C) after 56 days.	[256]
Rainbow trout (*Oncorhynchus mykiss*)	*Bacillus velezensis* MVCR2/ *Latilactobacillus sakei* RDB2	1 × 10^7^ CFU/g feed; 30 days	*B. velezensis* MVCR2 ↑ renal *tlr3* expression (~0.95 vs. ~0.45 C) and splenic *myd88* expression (~0.75 vs. ~0.53 C); *L. sakei* RDB2 ↑ renal *tlr9* expression (~0.055 vs. ~0.025 C) and *irak4* expression (~0.95 vs. ~0.68 C) after 10 days.	[257]

Note: ↑: increased; ↓: decreased; EPM: effective probiotic microorganisms; ALP: alkaline phosphatase; ACP: acid phosphatase; NBT: nitroblue tetrazolium reduction assay (oxidative burst); LYZ: lysozyme; AKP: alkaline phosphatase.

**Table 4:** Effects of probiotics on the resistance of food and ornamental fish species to various pathogens.

Fish Species	Probiotic	Dose and Duration of Administration	Main Effects	Reference
**Bacterial pathogens**				
Red tilapia (*Oreochromis niloticus* × *O. mossambicus*)	*Pediococcus acidilactici* CNCM I-4622—BACTOCELL^®^	1–3 g/kg feed; 63 days	↓ mortality after *Aeromonas hydrophila* challenge (70% in C vs. 45%, 35%, and 30%), dose-dependent	[258]
Nile tilapia (*Oreochromis niloticus*)	*Bacillus velezensis* BV0505-11	1 × 10^8^ CFU/g feed; 56 days	↑ survival after *Streptococcus agalactiae* challenge (77.31% vs. 47.96% in C); RPS = 56.55%	[259]
Red seabream (*Pagrus major*)	*Bacillus* sp. PM8313	1 × 10^8^ CFU/g feed; 8 weeks	↑ survival after *Edwardsiella tarda* challenge (66.67% vs. 0% in C)	[260]
Rainbow trout (*Oncorhynchus mykiss*)	*Bacillus subtilis* ATCC 6633	1 × 10^7^ CFU/g feed; 60 days	↑ survival after *Yersinia ruckeri* challenge (73.33% vs. 45.33% in C); RPS = 51.22%	[261]
Milkfish (*Chanos chanos*)	*Pediococcus pentosaceus* HLAB22	1 × 10^6^ CFU/g feed; 12 days	↑ survival after *Vibrio harveyi* challenge (83.33% vs. 33.33% in C)	[262]
Common carp (*Cyprinus carpio*)	*Enterobacter asburiae* E7	1 × 10^7^ CFU/g feed; 28 days	↑ survival after *Aeromonas veronii* challenge (91.05% vs. 54% in C)	[263]
Nile tilapia (*Oreochromis niloticus*)	*Bacillus subtilis*, *B. licheniformis* and *B. pumilus*	1 × 10^6^ or 2 × 10^6^ CFU/g feed; 10 weeks	↓ mortality after *Streptococcus iniae* challenge (6.67% at the highest dose vs. 81.67% in C)	[264]
Rainbow trout (*Oncorhynchus mykiss*)	*Lactobacillus delbrueckii* subsp. *bulgaricus*	5 × 10^7^ CFU/g feed; 60 days	↓ mortality after *Lactococcus garvieae* challenge (~26.6% vs. 73.3% in C)	[265]
Flathead grey mullet (*Mugil cephalus*)	*Lacticaseibacillus rhamnosus* FS3051	1 × 10^9^ CFU/g feed; 28 days	↑ survival after *Nocardia seriolae* challenge (70% vs. 35% in C)	[266]
Atlantic cod (*Gadus morhua*)	*Carnobacterium divergens* Lab01 and Lab19	Feeding with *Artemia franciscana* enriched with ~1 × 10^7^ CFU/mL; administered at 30, 35, 40, and 50 days post-hatch	↑ survival after *Vibrio anguillarum* challenge (~70% vs. ~30% in C)	[267]
**Viral pathogens**				
Red hybrid tilapia (*Oreochromis* spp.)	*Bacillus subtilis*, *B. licheniformis* and *B. pumilus* (Secure Yield)	0.5% or 1% in feed, equivalent to 1 × 10^7^ or 2 × 10^7^ CFU/g; 21 days	↓ mortality after tilapia lake virus (TiLV) challenge (25% and 24% vs. 32% in C); the 1% dose also reduced viral load	[268]
Hulong hybrid grouper (*Epinephelus fuscoguttatus* ♀ × *E. lanceolatus* ♂)	*Bacillus subtilis* 7k	1 × 10^6^, 1 × 10^8^, or 1 × 10^10^ CFU/g feed; 5 weeks	↓ mortality after Singapore grouper iridovirus (SGIV) challenge (46.67% and 30% vs. 73.33% in C), in a dose-dependent manner	[269]
Largemouth bass (*Micropterus salmoides*)	*Bacillus velezensis* Bv	1–3 × 10^8^ CFU/g feed; 28 days	↓ mortality after largemouth bass virus (LMBV) challenge (~40% vs. ~60% in C); ↓ viral load and severity of clinical signs.	[270]
Giant grouper (*Epinephelus lanceolatus*)	*Pediococcus pentosaceus* LAB4012	1 × 10^8^ CFU/g feed; 4 weeks	↓ mortality after nervous necrosis virus (NNV) challenge (66.7% vs. 80% in C); ↓ mortality after grouper iridovirus (GIV) challenge (46.7% vs. 86.7% in C)	[271]
Gibel carp (*Carassius auratus gibelio*)	*Bacillus subtilis* SY01 *Bacillus amyloliquefaciens* SY02	1 × 10^7^ CFU/g feed; 30 days	↑ survival after cyprinid herpesvirus 2 (CyHV-2) challenge (66.7% with SY01 and 54.2% with SY02 vs. 20.8% in C)	[272]
Gibel carp (*Carassius auratus gibelio*)	*Clostridium butyricum* Cb-F1610	1 × 10^4^ CFU/g body weight in feed and 1 × 10^6^ CFU/L in water; 1 week	↑ survival after Carassius auratus herpesvirus (CaHV) challenge (45% vs. 11% in C); ↓ viral load;	[273]
Orange-spotted grouper (*Epinephelus coioides*)	*Shewanella* sp. 0409	Live bacterial suspension sprayed onto the feed at a ratio of 37:100; feed administered at 4% of body weight; 2 months	↓ morbidity after nervous necrosis virus (NNV) challenge (10% vs. 100% in C); mortality 0% vs. 25%	[274]
Golden Pompano (*Trachinotus ovatus*)	*Bacillus amyloliquefaciens* E35	2 × 10^8^ CFU/g feed; 40 days	↓ mortality after Singapore grouper iridovirus (SGIV) challenge (38.1% vs. 55% in C); ↓ viral load	[275]
Japanese seabass (*Lateolabrax japonicus*)	*Lactococcus lactis* M48	1 × 10^6^/10^7^/10^8^ CFU/g feed; 28 days	↑ survival after sea perch iridovirus (SPIV) challenge (90% vs. 60% in C); ↓ viral replication	[276]
Rainbow trout (*Oncorhynchus mykiss*)	*Lactococcus lactis* NZ3900, without recombinant vector	1 × 10^8^/10^10^ CFU/g feed; days 1–7 and 15–21	↓ mortality after viral hemorrhagic septicemia virus (VHSV) challenge (47–50% vs. 64% in C); relative protection of 22–27%	[277]
**Parasitic and fungal agents**				
Rainbow trout (*Oncorhynchus mykiss)*	*Aeromonas sobria GC2*; *Brochothrix thermosphacta BA211*	GC2: 1 × 10^8^ cells/g feed; BA211: 1 × 10^10^ cells/g feed; 14 days	↑ survival after *Ichthyophthirius multifiliis* challenge (100% with GC2 vs. 2% with BA211 and 0% in C)	[278]
Striped catfish (*Pangasius hypophthalmus*)	*Lactobacillus plantarum* FNCC 226	2.3 × 10^5^, 4.0 × 10^5^ or 7.7 × 10^5^ CFU/mL; 5 days	At 7.7 × 10^5^ CFU/mL: ↓ *S. parasitica* infection diameter after 5 days (8.96 vs. 14.23 mm C) at the highest pathogen concentration tested (4 × 10^7^ zoospores/mL).	[279]
Guppy (*Poecilia reticulata*)	*Saccharomyces cerevisiae* *Bacillus licheniformis* *Bacillus latrospore*	0.5–1.5% *S. cerevisiae* + 1 × 10^9^ CFU *Bacillus* spores/100 g feed; 30 days	↑ survival after *I. multifiliis* challenge with 1.5% *S. cerevisiae* (100% vs. 50% in C); no parasites detected	[280]
Goldfish (*Carassius auratus*)	*Lactobacillus plantarum* NC8 (expressing IAG-52X from *I. multifiliis*)	1 × 10^6^ or 1 × 10^8^ CFU/g feed; 4 weeks	moderate *I. multifiliis* infection compared with severe infection in C; ↑ survival (50% and 60% vs. 40% in C)	[281]
Grass carp (*Ctenopharyngodon idella*)	*Bacillus subtilis* WB600 (expressing CsCP from *C. sinensis*)	1 × 10^7^ CFU/g feed; 8 weeks	No fish tested positive for *Clonorchis sinensis*, compared with 27% infected and two mortalities in C	[282]
Pirarucu (*Arapaima gigas*)	*Enterococcus faecium*	1 × 10^6^/1 × 10^8^ CFU/g feed; 68 days	At 1 × 10^8^ CFU/g feed: ↓ *Trichodina* sp. prevalence in mucus (50% vs. 100% C) and mean parasite intensity in mucus (40 vs. 102 C) and gills (8.0 vs. 32.3 C) after 68 days.	[283]
Nile Tilapia (*Oreochromis niloticus*)	Commercial product EMS: lactic acid bacteria + *Saccharomyces*	2% or 4% in feed; 21 days	2%: ↓ *T. compacta* infection rate (20% vs. 46% C at 15 days; 28% vs. 85% C at 21 days) and infection intensity (≤1 vs. >5 parasites/fish C at 15 days); ↓ cumulative mortality (16% vs. 53% C).	[284]
Grass carp (*Ctenopharyngodon idella*)	*Bacillus subtilis* (expressing CsPmy from *C. sinensis*)	1 × 10^5^/1 × 10^8^/1 × 10^11^ CFU/g feed; 6 weeks	At 1 × 10^11^ CFU/g feed: ↓ *C. sinensis* metacercarial load after challenge (7.2 vs. 13.2 metacercariae/g fish flesh C).	[285]
Goldfish (*Carassius auratus*)	*Bacillus subtilis* IBRC-M 10997	1 × 10^9^ CFU/g feed; 80 days	1 × 10^9^ CFU/g feed: ↓ *I. multifiliis* trophont intensity (2.81 and 2.85 vs. 17.81 C, respectively) 5 days after challenge; ↓ skin and gill lesions.	[152]
Spotted snakehead (*Channa punctatus*)	*Saccharomyces cerevisiae*	2.5 g/kg feed 30 days before infection and 8 weeks after infection	↓ mortality after *Aphanomyces invadans* challenge (15% vs. 85% in C)	[286]

Note: The arrows ↑ and ↓ indicate increased and decreased, respectively; C: Control.

**Table 5:** Effects of probiotics on hematological and biochemical parameters in farmed fish.

Fish Species	Probiotic	Dose and Duration of Administration	Main Effects	Reference
Gilthead seabream (*Sparus aurata*)	*Bacillus subtilis* + *B. licheniformis* (SANOLIFERPRO-W)	0.01, 0.02 or 0.03 g/m^3^ water; 10 weeks	0.03 g/m^3^ water: ↑ Ht (35.92% vs. 28.81% C), RBC (2.72 vs. 2.34 × 10^6^/µL C), Hb (11.75 vs. 8.87 g/dL C), total protein (5.32 vs. 2.95 g/dL C), and albumin (2.97 vs. 1.58 g/dL C), reflecting an improved hematological profile and enhanced blood oxygen-carrying capacity.	[287]
Tilapia (*Oreochromis niloticus*)	*Bacillus subtilis* BSN100	1 × 10^6^/10^8^/10^10^ CFU/g feed; 8 weeks	1 × 10^10^ CFU/g feed: ↑ Ht (38.62% vs. 27.31% C), Hb (10.8 vs. 7.2 g/dL C), and MCHC (30.7 vs. 26.5 g/dL C); ↓ AST (9.67 vs. 15.94 U/L C) and ALT (24.02 vs. 37.12 U/L C), suggesting an improved hematological profile.	[288]
Common carp (*Cyprinus carpio*)	*Enterococcus casseliflavus* EC-001	1 × 10^7^, 1 × 10^8^ or 1 × 10^9^ CFU/g feed; 8 weeks	At 1 × 10^9^ CFU/g feed: ↑ RBC (26.21 vs. 21.6 × 10^5^ cells/mm^3^ C), Ht (22.8% vs. 19.5% C), Hb (8.26 vs. 7.22 g/dL C), total protein (4.0 vs. 3.3 g/dL C), and albumin (1.43 vs. 1.02 g/dL C), indicating improved hematological parameters and increased serum protein levels.	[289]
Hybrid sturgeon (*Acipenser baerii* ♀ × *A. schrenckii* ♂)	*Bacillus licheniformis*	0.10%, 0.20% or 0.40% feed; 120 days	The dose of 0.20% showed the most favorable results ↑ total cholesterol (3.87 vs. 2.42 mmol/L C), triglycerides (9.22 vs. 7.70 mmol/L C), AST (677.81 vs. 556.41 U/L C) and ALT (8.07 vs. 7.02 U/L C); total protein and ALP showed no significant changes.	[290]
Beluga sturgeon (*Huso huso*)	*Pediococcus acidilactici* MA18/5M (Bactocell^®^)	1 × 10^7^, 1 × 10^8^ or 1 × 10^9^ CFU/g feed; 8 weeks	At 1 × 10^9^ CFU/g feed: ↑ total protein (2.52 vs. 1.89 g/dL C) and albumin (1.71 vs. 1.21 g/dL C), but also ↑ ALT (21.68 vs. 10.63 IU/dL C) and AST (227.11 vs. 129.18 IU/dL C), indicating increased serum protein levels but possible adverse effects at the highest dose.	[291]
Rainbow trout (*Oncorhynchus mykiss*)	*Lactococcus lactis* subsp. *lactis* PTCC 1403	1 × 10^9^, 2 × 10^9^ or 3 × 10^9^ CFU/g feed; 8 weeks	At 1 × 10^9^ CFU/g feed: ↑ Ht (52.33% vs. 42.67% C); at 2 × 10^9^ CFU/g feed: ↓ glucose (35.2 vs. 56.3 mg/dL C); showing a moderate and parameter-specific response.	[292]
Rohu (*Labeo rohita*)	*Saccharomyces cerevisiae*	1, 2 or 4 g/kg feed; 90 days	At 4 g/kg feed: ↑ Hb (12.98 vs. 11.45 g/dL C) and RBC (1.63 vs. 1.46 × 10^6^/mm^3^ C), suggesting an improved erythrocyte profile; ↑ blood glucose (101.50 vs. 81.00 mg/dL C).	[293]
Striped catfish (*Pangasianodon hypophthalmus*)	Sanolife PRO-F: *Bacillus subtilis*, *B. licheniformis* and *B. pumilus*	0.2 g/kg feed; administered during the larval–fingerling stage for 20 days and/or during the fingerling–juvenile stage for 45 days; subsequent evaluation over 90 days without probiotic	At 0.2 g/kg feed: ↑ RBC (1.49 vs. 0.59 × 10^6^/mm^3^ C) and WBC (1.64 vs. 0.75 × 10^5^/mm^3^ C) after phase 2; these effects persisted during the probiotic-free grow-out phase, with ↑ Hb (4.05 vs. 3.10 g/dL C), RBC (3.97 vs. 1.31 × 10^6^/mm^3^ C), and WBC (1.89 vs. 0.79 × 10^5^/mm^3^ C), reflecting a sustained improvement in hematological status.	[294]
Asian seabass (*Lates calcarifer*)	Three mixtures composed of *Lactiplantibacillus plantarum* strains, alone or combined with *L. rhamnosus*, *L. acidophilus*, *Lactobacillus delbrueckii subsp. bulgaricus*, *Bacillus thuringiensis* QQ1, and *B. cereus* QQ2	mixtures with a total count of 3.3 × 10^10^ CFU/mL; continuous or cyclic administration, 14 days with probiotic/14 days without; 16 weeks	The probiotic mixtures increased Hb and Ht (CF-II: 15.2 vs. 5.1 g/dL C; 29.3% vs. 19.0% C), while pulse-feeding also increased RBC (PF-II: 2.5 vs. 1.6 × 10^6^/µL C), reflecting an improved hematological profile.	[295]
Stinging catfish (*Heteropneustes fossilis*)	Autochthonous consortium of *Bacillus* *safensis* HF8, *B. altitudinis* HF10, *B. pacificus* HF19, *B. cereus* HF20, and *B. cereus* HF21	1.15 × 10^9^–2.30 × 10^9^ CFU/kg feed; 60 days	At 60 days: ↑ Hb (12.81 vs. 10.80 g/dL C), RBC (2.36 vs. 1.83 × 10^6^/mm^3^ C), and Ht (38.42% vs. 32.4% C); the consortium mainly improved the erythrocyte component and oxygen-carrying capacity.	[296]
Hybrid yellow catfish (*Pelteobagrus fulvidraco* ♀ × *P. vachelli* ♂)	*Bacillus velezensis* YFI-E109	0.90 × 10^8^–0.83 × 10^12^ CFU/kg feed; 6 weeks	At intermediate doses: ↑ TP (33.30 vs. 26.36 g/L C) and ALB (8.24 vs. 6.49 g/L C); ↓ TG at most doses (3.32 vs. 5.40 g/L C), while higher doses ↓ GLU (3.27 vs. 5.62 mmol/L C), showing dose-dependent changes in serum protein, triglyceride, and glucose levels.	[297]
Fringed-lipped peninsula carp (*Labeo fimbriatus*)	*Bacillus subtilis* MTCC-121	1 × 10^4^ or 1 × 10^6^ CFU/g feed; 60 days	In the groups given the probiotic alone: the dose of 1 × 10^6^ CFU/g feed showed moderate results: ↑ Hb (~5.4 vs. ~4.1 g/dL C), total protein (~4.3 vs. ~3.7 g/dL C), and globulin (~2.7 vs. ~2.2 g/dL C), indicating an improved hematological and serum protein profile.	[298]

Note: C: Control; ALB: albumin; ALP: alkaline phosphatase; ALT: alanine aminotransferase; AST: aspartate aminotransferase; CFU: colony-forming units; GLU: glucose; Hb: hemoglobin; Ht: hematocrit; MCHC: mean corpuscular hemoglobin concentration; QQ: quorum quenching; RBC: red blood cells; TG: triglycerides; TP: total protein; WBC: white blood cells; CF-II: continuous feeding with probiotic mixture II; PF-II: pulse-feeding with probiotic mixture II; ↑: increased; ↓: decreased.

**Table 6:** Effects of probiotics in attenuating various stress factors in farmed fish.

Probiotic	Fish Species	Dose and Duration of Administration	Stress Factor	Main Effects	Reference
*Bacillus subtilis* DSM 32315	Nile tilapia (*Oreochromis niloticus*)	0.1–0.3% of the feed, 8 weeks	Acute ammonia exposure: 43.61 mg/L total ammonia nitrogen, 48 h	The 0.3% dose showed the most promising results: ↓ mortality (8.75% vs. 25.00% C), ↑ T-AOC (0.54 vs. 0.39 U/mg protein C), and ↓ MDA (0.98 vs. 1.46 nmol/mg protein C), indicating improved antioxidant defense and stress tolerance.	[308]
*Aspergillus oryzae*	Nile tilapia (*Oreochromis niloticus*)	1 × 10^6^ or 1 × 10^8^ CFU/g feed, 60 days	Hypoxia: reduction in dissolved oxygen from 7.41 to 3.23 mg/L	The dose of 1 × 10^8^ CFU/g indicated a favorable response: ↓ cortisol (~0.68 vs. ~0.93 μM/mL C), ↑ SOD (~26 vs. ~21 IU/L C), and ↑ GPX (~26 vs. ~16 IU/L C), indicating improved antioxidant defense and hypoxia stress tolerance.	[309]
*Bacillus subtilis* E221	Nile tilapia (*Oreochromis niloticus*)	1 × 10^8^ CFU/g feed, 8 weeks	High salinity: 16 psu	↑ WG (~1290% vs. ~1100% C), ↓ FCR (~1.20 vs. ~1.33 C), and ↑ CH50 (~72 vs. ~57 U/mL C), indicating improved growth and immune status under hypersaline stress (16 psu).	[310]
*S. cerevisiae*; mixture of *B. subtilis*, *B. megaterium*, *B. licheniformis*	Nile tilapia (*Oreochromis niloticus*)	5.6 × 10^8^ and 1 × 10^6^ CFU/g preparation, respectively; 0.5 mL/L water, weekly, for 120 days	4 h Transport	The dose of 1 × 10^6^ CFU/g showed the strongest overall response to transport stress: ↓ cortisol (~14 vs. ~24 μg/dL C) immediately after transport and ↓ MDA (~52 vs. ~76 μmol/L C) after 24 h, together with lower AST and ALT, indicating improved transport stress tolerance and reduced oxidative stress.	[311]
*Bacillus coagulans* SCC-19	Common carp (*Cyprinus carpio*)	1 × 10^8^ CFU/g feed, 60 days	Waterborne cadmium: 0.5 mg/L	↓ cadmium accumulation in the liver(~6 vs. ~29 μg/g wet weight C) and kidney (~4.5 vs. ~21 μg/g wet weight C); while improving antioxidant capacity (SOD: ~170 vs. ~100 U/mg protein C; MDA: ~13 vs. ~23 nmol/mg protein C) and nonspecific immunity, thereby attenuating Cd toxicity.	[312]
AquaStar^®^ Growout: mixture of *Bacillus*, *Enterococcus*, *Lactobacillus* and *Pediococcus*	Nile tilapia (*Oreochromis niloticus*)	3 g/kg feed, 21 days: 7 days of exposure and 14 days of recovery	Malathion: 0.06 mg/L	↓ mortality from 53.3% to 26.7%; ↓ MDA(~8.5 vs. ~11.5 nmol/mL C), with lower ALT and AST, suggesting reduced oxidative damage and improved physiological resilience.	[313]
*Rhodobacter sphaeroides* SC01	Common carp (*Cyprinus carpio*)	1 × 10^8^ CFU/g feed, 50 days	Lead: 1 mg/L	↓ Pb accumulation in liver (~2.4 vs. ~7.0 mg/kg C; 65.34% reduction and kidney (~2.3 vs. ~5.8 mg/kg C; 60.38% reduction), together with ↓ hepatic MDA by 48.52%, resulting in alleviation of Pb-induced oxidative stress.	[314]
*Lactococcus lactis* 33HT	African catfish (*Clarias gariepinus*)	1 × 10^9^ or 1 × 10^10^ CFU/g feed, 60 days	Mercury: 0.13 mg/L	1 × 10^10^ CFU/g feed: ↑ RBC (2.94 vs. 2.31 × 10^6^/mm^3^ C) and lysozyme (~17.5 vs. ~11 U/L C); ↓ AST (~18 vs. ~21 U/L C) and muscle Hg accumulation (~1.1 vs. ~2.6 mg/kg C), with 100% survival vs. 90% C, resulting in improved hematological and immune status alongside reduced Hg toxicity.	[315]
*Bacillus coagulans* *Clostridium butyricum*	Goldfish (*Carassius auratus*)	1 × 10^6^ CFU/g feed, 14 days	Glyphosate: 1 μg/L and 1 mg/L	*C. butyricum* ↑ SGR (3.48 vs. 1.22%/d C), while both probiotics ↓ intestinal GLY accumulation (60.6–63.3 vs. 303 ng/g dry weight C) and restored gut microbiota and metabolite profiles, indicating attenuation of GLY-induced growth retardation and intestinal toxicity.	[316]
Mixture of *B. subtilis*, *B. thuringiensis*, *L. plantarum* and *L. buchneri*	Rohu (*Labeo rohita*)	1 mL/L water, administered every two days, for 42 days	Chromium: 4 mg/L	↑ WG (2.44 vs. 1.39 g C), ↓ FCR (1.77 vs. 3.21 C) and mortality (10% vs. 20% C); Hb (~9.5 vs. ~7.4 g/dL C) and glucose (~64 vs. ~80 mg/dL C) were restored toward normal levels, with ↓ erythrocyte abnormalities, indicating improved tolerance to Cr-induced stress.	[317]
*Lactobacillus rhamnosus* GG	Zebrafish (*Danio rerio*)	1 × 10^8^ CFU/g feed, 28 days	Microcystin-LR: 2.2 and 22 μg/L	↓ brain MCLR accumulation (females: 0.35 vs. 1.10 ng/g C; males: 0.17 vs. 1.04 ng/g C), restored swimming speed (~4.2 vs. ~3.2 cm/s in females; ~4.1 vs. ~3.0 cm/s in males), and ↓ cortisol (~80 vs. ~125 ng/mL C in males), indicating attenuation of MCLR-induced neurotoxicity and HPI-axis stress.	[318]
Mixture of *B. subtilis*, *L. plantarum* and *L. buchneri*	Striped catfish (*Pangasianodon hypophthalmus*)	1 mL/L water, for 42 days; reapplied after the weekly water change	Fenitrothion (Sumithion^®^) 0.6 mg/L	Antioxidant-related gene expression shifted toward control levels (*SOD*: ~1.1 vs. ~0.6 C; *GPx*: ~1.5 vs. ~3.4 C), ↓ glucose (~130 vs. ~150 mg/dL C), and improved intestinal integrity (villus length: 97.92 vs. 54.49 μm C), resulting in the attenuation of pesticide-induced oxidative and physiological stress.	[319]
*B. licheniformis* + *B. amyloliquefaciens*	Snook larvae *(Centropomus undecimalis)*	1:1 mixture; target concentration of 1.1 × 10^5^ CFU/mL in water, administered in the water and in live feed throughout the larval rearing period, for 26–28 days	Larval transport	↑ survival immediately after transport (~90–98% vs. ~78–85% C) and 7 days post-transport (~85–95% vs. ~65–68% C), indicating improved resistance to transport stress.	[320]
*Lactobacillus acidophilus* PTCC 1608	Common carp (*Cyprinus carpio*)	1.5 × 10^7^ or 3 × 10^7^ CFU/g feed, for 60 days	Stress factor: crowding stress: 80% reduction in water volume for 6 h	After crowding stress, ↓ MDA (39.13 vs. 45.23 nmol/mL C) and ↓ cortisol (100.26 vs. 110.66 ng/mL C), indicating reduced oxidative and physiological stress.	[321]
Mixture of *Bacillus subtilis*, *Lactobacillus plantarum* and *L. buchneri*	Rohu (*Labeo rohita*)	1 mL/L water, administered every two days, for 42 days	High temperature: 33 and 36 °C	Restored Hb (~11.2 vs. ~8.8 g/dL C), markedly ↓ erythrocyte abnormalities, and ↓ *hsp70/hsp90* expression (~1.0/~1.15 vs. ~2.5/~2.05 C), indicating attenuation of heat-induced physiological and cellular stress	[322]

Note: C: Control; T-AOC: Total Antioxidant Capacity; ALT: alanine aminotransferase; AST: aspartate aminotransferase; GPx: glutathione peroxidase; hsp: heat shock protein; MDA: malondialdehyde; SOD: superoxide dismutase; ↑: increased; ↓: decreased.

**Table 7:** Effects of different probiotics on water quality parameters in aquaculture systems.

Probiotic	Species and Rearing System	Dose and Duration of Administration	Water Parameters Assessed	Main Effects	Reference
*Bacillus* *amyloliquefaciens*	Polyculture of grass carp (*Ctenopharyngodon idella*), Prussian carp (*Carassius gibelio*), and silver carp (*Hypophthalmichthys molitrix*); 300 L tanks Fish-stocked system (14 fish/tank)	1 × 10^11^ CFU/m^3^, for 7 days; 15 days	Ammonia, nitrite, nitrate, total nitrogen, total phosphorus, COD, pH, and dissolved oxygen	↓ ammonia by 41.6–50.7%; ↓ total nitrogen by 49%; ↓ total phosphorus by 21.8–30.9%; ↓ COD by 15–20%; ↑ nitrite, compared with controls	[330]
Prozym Powder Ultra^®^, Microban Aqua^®^, AquaStar^®^, and Sanolife PRO-W^®^; mixtures of *Bacillus* spp., lactic acid bacteria, yeasts, and other bacteria	Nile tilapia (*Oreochromis niloticus*); 20 m^3^ concrete ponds, zero-water-exchange system Fish-stocked (150 fish/tank)	0.001–0.002 g/m^3^/day, depending on the product; 8 weeks	Ammonia, nitrite, nitrate, pH, dissolved oxygen; *Vibrio* spp. and *Bacillus* spp. in the water	↓ ammonia, nitrite, and nitrate; ↓ *Vibrio* spp. counts; ↑ *Bacillus* spp. counts in the water	[331]
*Bacillus* *licheniformis* VLPPro^®^ SB538	Hybrid grouper (*Epinephelus fuscoguttatus* ♀ × *E. polyphekadion* ♂); 200 L seawater tanks Zero-water-exchange system Fish-stocked (20 fish/tank)	Applied to the water every 3 days; 22 days	Ammonia, nitrite, nitrate, phosphate, and pH	Significant ↓ in ammonia in all treated groups; significant ↓ in nitrite at the 0.375 and 0.750 g/m^3^ doses	[332]
Compound microbial agent: yeast, *Bacillus subtilis*, and lactic acid bacteria, in a 1:1:1 ratio	Largemouth bass (*Micropterus salmoides*); 100 L aquaria, RAS with biological filter Fish-stocked (60 fish/aquarium)	4 g added to the feed every 7 days; 8 weeks	pH, dissolved oxygen, ammonia nitrogen, nitrite, total nitrogen, and total phosphorus	↓ ammonia nitrogen by 41.75%; ↓ nitrite by 19.35%; ↓ total nitrogen by 22.45%; ↓ total phosphorus by 9.04%; ↑ dissolved oxygen by 4.63%; delayed increase in pH	[333]
Compound commercial probiotic, including *Lactobacillus*, *Acidobacteriota*, and other bacteria	Whitespotted conger *(Conger myriaster)*; industrial RAS with 25 m^3^ tanks Fish-stocked system (stocking density: 10.00 ± 2.15 kg/m^3^)	1 × 10^9^ CFU/mL; 8 mL/m^3^, once every 7 days; 70 days	Ammonia nitrogen, nitrite, nitrate, total nitrogen, total phosphorus, phosphate, and COD	Significant ↓ in ammonia nitrogen, nitrite, nitrate, total nitrogen, and COD; ↑ total phosphorus and phosphate removal rates.	[334]
*Lactiplantibacillus plantarum* *Bacillus toyonensis*	Nile tilapia (*Oreochromis niloticus*); 75 L aquaria Fish-stocked system (10 fish/aquarium)	1 mL/L; 70 days	Ammonia, nitrite, nitrate, pH, dissolved oxygen, and bacterial load of the water	↓ ammonia and nitrite; ↑ nitrate with the individual probiotic treatments; no significant changes in pH or dissolved oxygen; ↓ total coliforms, *E. coli*, and *Salmonella* spp. in the rearing water	[335]
*Bacillus velezensis* AP193	Channel catfish (*Ictalurus punctatus*); 0.04 ha ponds Fish-stocked system (~861 fish/pond)	4 × 10^7^ CFU/g feed; 10 weeks	Total ammonia nitrogen, nitrite, nitrate, total nitrogen, and total phosphorus	↓ total phosphorus by 19%; ↓ total nitrogen by 43%; ↓ nitrate by 75%; no significant changes in total ammonia nitrogen or nitrite	[336]
pH FIXER, commercial product based on *Bacillus* sp.	Nile tilapia (*Oreochromis niloticus*); ponds, Fish-stocked system (stocking density: 400 fish/decimal)	1 kg/ha, weekly; 75 days	Dissolved oxygen, free ammonia, pH, alkalinity, and temperature	↑ dissolved oxygen to 6.79 ± 0.19 mg/L; ↓ free ammonia to 0.018 ± 0.002 mg/L; no significant change in pH	[337]
*Bacillus subtilis* COFCAU_BSP3	Rohu (*Labeo rohita*); 60 L aquaria, 15 fish per aquarium Fish-stocked system	1 × 10^8^ CFU/mL water, administered on days 0, 10, and 20; 30 days	Dissolved oxygen, alkalinity, hardness, pH, ammonia nitrogen, nitrite, and nitrate	↓ ammonia nitrogen by 50%; ↓ nitrite by 52.63%; ↑ nitrate by 1.9-fold; dissolved oxygen, pH, alkalinity, and hardness showed no significant changes	[338]
Commercial EM preparation: *Lactobacillus*, *Saccharomyces*, photosynthetic bacteria, *Bacillales*	Japanese grenadier anchovy *(Coilia nasus)*; 160 m^3^ ponds Fish-stocked system (300 fish/pond)	Preparation with a concentration of 1 × 10^8^ CFU/g, applied to the water every 4 days; 120 days	Ammonia nitrogen, nitrite, nitrate, total nitrogen; microbial community and genes involved in the nitrogen cycle	↓ ammonia nitrogen and nitrite; ↓ total nitrogen; ↑ nitrate; ↑ relative abundance of nitrification-related genes (*amoC_B, hao*) and denitrification-related genes (*nirS, nosZ*); changes in the microbial community of the water	[339]

Note: ↑: increased; ↓: decreased; COD: chemical oxygen demand; EM: effective microorganisms; RAS: recirculating aquaculture system.

**Table 8:** Main types of prebiotics and compounds with prebiotic potential investigated in aquaculture.

Prebiotic	Description	Reference
Fructo-oligosaccharides (FOS)	FOS are fructose-based oligosaccharides, with scFOS representing the short-chain forms. They occur naturally in fruits, vegetables, and cereals. They are obtained commercially through the hydrolysis of inulin or by enzymatic/microbial synthesis from sucrose.	[361]
Inulin	Inulin is a non-digestible fructan composed of predominantly linear chains of fructose units linked by β-(2→1) bonds. It is found in chicory, Jerusalem artichoke, garlic, onion, and asparagus. It is obtained by extraction from plant sources, most commonly using hot water.	[362]
Mannan-oligosaccharides (MOS)	MOS are non-digestible short-chain oligosaccharides composed of mannose. They are obtained through the hydrolysis of mannans from plant sources or from the cell wall of the yeast *S. cerevisiae*.	[363]
β-glucans (BG)	BG are polysaccharides composed of glucose units linked by β-(1→3) and β-(1→6) bonds. They are isolated from the cell wall of the yeast S. cerevisiae by physical, chemical, and/or enzymatic methods.	[364]
Galacto-oligosaccharides (GOS)	GOS are non-digestible oligosaccharides obtained from milk lactose through transgalactosylation catalysed by β-galactosidase.	[365]
Xylo-oligosaccharides (XOS)	XOS are xylose oligomers linked by β-(1→4) bonds, obtained from the xylan of lignocellulosic biomass through autohydrolysis, or chemical or enzymatic hydrolysis.	[353]
Arabinoxylan-oligosaccharides (AXOS)	AXOS are non-digestible oligosaccharides derived from cereal arabinoxylan. They are obtained through the enzymatic hydrolysis of arabinoxylan with xylanases.	[366]
Isomalto-oligosaccharides (IMO)	IMO are oligosaccharides composed of glucose units linked predominantly by α-(1→6) bonds. They are obtained enzymatically from starch.	[367]
Chitin	Chitin is a natural polysaccharide found mainly in the shells of crustaceans, but also in insects and fungi. It is usually obtained through chemical extraction from crustacean waste.	[355]
Chitosan	Chitosan is a cationic polysaccharide derived from chitin. It is obtained through the deacetylation of chitin.	
Chito-oligosaccharides (COS)	COS are oligomers derived from chitosan. They are obtained through chemical or enzymatic processes.	
Fucoidan	Fucoidan is a sulphated polysaccharide, rich in fucose, extracted from brown algae such as *Ascophyllum nodosum*, *Cladosiphon okamuranus*, *Fucus vesiculosus*, *Laminaria japonica*, and *Sargassum wightii*. It can be obtained through extraction with hot water, dilute acids, or alkalis.	[356]
Laminarin	Laminarin (LAM) is a β-glucan extracted from brown algae such as *Laminaria digitata*, *Laminaria japonica*, *Saccharina latissima*, and *Sargassum henslowianum*. It is obtained through extraction with hot water.	[368]
Alginate oligosaccharides (AOS)	AOS are low-molecular-weight products resulting from the degradation of alginate, an anionic polysaccharide frequently isolated from brown algae such as *Laminaria* and *Macrocystis*. They are obtained through enzymatic degradation, acid hydrolysis, or oxidative degradation.	[369]
Pectic oligosaccharides (POS)	Pectic oligosaccharides (POS) are oligosaccharides obtained through the hydrolysis of pectin, a plant polysaccharide present in fruits, vegetables, and numerous agricultural by-products. They are produced by enzymatic or chemical methods, or by emerging technologies (ultrasound/microwave).	[370]

**Table 9:** Effects of prebiotic administration on growth performance and intestinal health in farmed fish.

Prebiotic	Fish Species	Dose and Duration of Administration	Main Effects	Reference
MOS	Grass carp (*Ctenopharyngodon idella*)	200–1000 mg/kg feed; 60 days	At 400 mg/kg MOS vs. control: ↑ PWG (296.3% vs. 225.6% C) and SGR (2.29% vs. 1.97% C); ↓ *A. hydrophila* (6.85 vs. 7.46 C) ^a^ and *E. coli* (6.75 vs. 7.42 C); improved intestinal integrity and antioxidant capacity. Growth responses declined at higher doses, with 1000 mg/kg resulting in lower PWG and SGR than the control.	[378]
COS	Nile tilapia (*Oreochromis niloticus*)	0.4–1.2% of the feed; 8 weeks	↑ WG (13.86–16.45 g vs. 9.50 g C), SGR (2.09–2.51 vs. 1.51 C) and FE (0.72–0.78 vs. 0.66 C); ↑ protease, amylase, cellulase and lipase activities at 0.8–1.2% COS.	[379]
Fucoidan	Large yellow croaker (*Larimichthys crocea*)	0.5–2% of the feed; 30 days	↑ WGR (2945.55–3277.67% vs. 2270.87% C) and SGR (11.34–11.73 vs. 10.55 C); ↑ villus height at 1% Fuc (156.85 vs. 85.67 μm C); ↑ Bacteroidetes and ↓ opportunistic pathogens; at later developmental stages, ↑ lipase, AKP and LAP and ↓ α-amylase. Growth improved at all fucoidan levels, with no significant differences among doses, while intestinal morphology showed the strongest response at 1%.	[380]
Chitosan	Silver barb (*Barbonymus gonionotus*)	1–3 g/kg feed; 60 days	↑ weight gain (39.40 vs. 26.11 g C) and SGR (1.80 vs. 1.39%/day C); ↓ FCR (1.20 vs. 1.45 C); ↑ LAB (5.63 vs. 3.98 C) ^a^; ↑ villus length (~580 vs. ~330 μm C) and digestive enzyme activities. Higher doses produced weaker growth responses.	[381]
FOS	Japanese seabass (*Lateolabrax japonicus*)	0.5–6 g/kg feed; 28 days	At 1 g/kg FOS: ↑ WG (123.08% vs. 95.64% C) and SGR (2.86 vs. 2.39%/day C); ↓ FCR (0.95 vs. 1.20 C); ↑ erepsin activity (2753.1 vs. 1746.6 U/mg protein C). At 2 g/kg: ↑ intestinal lipase activity (69.83 vs. 55.37 U/mg protein C). Higher doses (4–6 g/kg) did not significantly improve growth.	[382]
Microencapsulated inulin	Striped catfish (*Pangasianodon hypophthalmus*)	0.4%, 0.8% and 1.2% of the feed; 8 weeks	At 0.8% inulin: ↑ FBW (35.47 vs. 29.80 g C), ADG (0.51 vs. 0.41 g/day C) and SGR (3.28 vs. 2.93%/day C); ↓ FCR (1.59 vs. 2.15 C); ↑ cellulase activity and intestinal villus height, width and goblet cell number. At 0.4%, growth parameters did not differ significantly from the control, whereas 1.2% significantly improved growth and FCR but provided no additional benefit over 0.8%.	[383]
β-glucan derived from algae	Asian swamp eel *(Monopterus albus)*	250–2000 mg/kg feed; 8 weeks	At 0.1% β-glucan: ↑ WGR (147.36% vs. 63.50% C), SGR (1.51 vs. 0.82%/day C); ↓ FCR (1.12 vs. 2.63 C); ↑ trypsin (2987.43 vs. 1322.68 U/mg protein C); ↑ amylase (37.88 vs. 25.27 U/mg protein C); ↑ lipase (336.36 vs. 281.23 U/mg protein C); ↑ intestinal villus length and microbial diversity. Growth improved at all tested doses, with the strongest response at 0.1% while the effect was lower at 0.2%.	[384]
GOS	Hybrid sturgeon (*Acipenser baerii* ♀ × *A. schrenckii* ♂)	1%, 3% and 5% of the feed; 8 weeks	At 3% GOS: ↑ WG (24.41 vs. 16.76 g C), SGR (1.52 vs. 1.16%/day C); ↓ FCR (1.11 vs. 1.37 C); ↑ intestinal villus and microvilli height and goblet cell number; improved intestinal morphology. The growth response increased from 1% to 3% GOS, but no further improvement was observed at 5%.	[385]

Note: PWG: Percent Weight Gain; C: Control; WG: weight gain; SGR: Specific Growth Rate; FCR: Feed conversion ratio; ADG: Average Daily Gain; AKP: alkaline phosphatase; LAP: leucine aminopeptidase; ↑: increased; ↓: decreased; ^a^ Bacterial counts are expressed as log CFU/g intestinal content; WGR: weight gain rate; LAB: lactic acid bacteria; FE: feed efficiency.

**Table 10:** Effects of prebiotics on the immune response and resistance to pathogens in fish.

Prebiotic	Fish Species	Dose and Duration of Administration	Main Effects	Reference
β-glucan GAS1	Rainbow trout (*Oncorhynchus mykiss*)	0.2 and 0.5%; 36 days	At 0.2%: ↑ plasma lysozyme activity at D15 and total Ig at D36; ↓ splenic *il-1β* expression after challenge; ↑ survival after *A. salmonicida* subsp. *achromogenes* challenge (43.3% vs. 16.7% C). Survival was also higher at 0.5% (30.0% vs. 16.7% C), but lower than at 0.2%.	[387]
XOS	Olive flounder (*Paralichthys olivaceus*)	1, 2 and 3%; 8 weeks	At 3% XOS: ↑ serum lysozyme activity (6.24 vs. 3.56 U/mL C) and antiprotease activity (39.3% vs. 20.2% C); ↑ survival after *E. tarda* challenge (~53% vs. ~3% C). Serum ACH50 activity was highest at 1% XOS (34.6 vs. 15.9 U/mL C) and decreased at 2–3%.	[388]
MOS	Pompano (*Trachinotus ovatus*)	0.1–0.8%; 8 weeks	At 0.2% MOS: ↑ WBC (0.75 vs. 0.50 × 10^5^ mm^−3^ C) and lymphocytes (66.20% vs. 48.72% C); ↑ PR, PI and serum lysozyme activity (~25% vs. C); ↑ survival after *S. iniae* challenge (~70% vs. ~10% C). The 0.1–0.6% doses also increased post-challenge survival, but the highest response was observed at 0.2%, while 0.8% did not significantly improve survival.	[389]
β-glucan	Zebrafish (*Danio rerio*)	0.01 and 0.025%; 14 days	0.025%: ↑ survival after SVCV challenge (~93% vs. ~71% C); ↑ splenic expression of type I IFN-related genes (*ifnj1*, *ifnj2*, *ifnj3*, *mxb*, *mxc*, *tlr7*, *rig1*, *mavs*, *irf3*, and *irf7*). The 0.01% dose also increased several IFN-related genes but did not significantly improve survival.	[390]
Poly-β-hydroxybutyrate (PHB)	Prussian carp *(Carassius auratus* *Gibelio)*	0.5, 1, 2, 4 and 8%; 30 and 60 days	4% for 30 days: ↑ splenic expression of immune-related genes (*KRT-8*, *MPO*, *DUSP-1*, *IL-11*, *ITLN*, and *PNP-5α*); ↓ cumulative mortality after CyHV-2 challenge (25% vs. 75% C), *A. hydrophila* challenge (50% vs. 62.5% C), and *A. veronii* challenge (12.5% vs. 50% C). The strongest immune gene response occurred at 4% after 30 days, whereas after 60 days several immune markers were highest at the lower 0.5% dose.	[391]
Mannose	Largemouth bass *(Micropterus salmoides*) Zebrafish (*Danio rerio*)	0.25, 0.5 and 1%; 14 days	At 0.5% mannose in zebrafish: ↑ survival after SVCV challenge (~40% vs. ~10% C) and ↑ type I IFN/ISG expression. In largemouth bass, survival after LMBV challenge was highest at 1% mannose (~63% vs. ~13% C).	[392]
cMOS	*Grass carp* *(Ctenopharyngodon idella*)	1, 2 and 4 g/kg; 6 weeks	4‰ cMOS for 3 weeks: ↓ *I. multifiliis* trophont burden and mortality (20% vs. 33.3% C); ↓ gill damage; ↑ gill mucous cells and T/NK cells before challenge. Similar protection was obtained with 2‰ cMOS after 4 weeks, whereas 1‰ did not produce a consistently significant effect	[393]
β-glucan	White sturgeon (*Acipenser transmontanus*)	0.1 and 0.3%; 21 days	At 0.3% β-glucan: ↓ mortality after *V. botryosa* challenge (~33% vs. ~52% C). At 0.1%: ↓ splenic fungal abundance (~1.6 vs. ~3.3 C) ^a^. Both doses ↑ splenic *IL-17* expression after challenge.	[394]
FOS + β-1,3-glucan	Nile Tilapia *(Oreochromis niloticus)*	0.5, 1 and 1.5 g/kg; 70 days	At 1.5 g/kg: ↑ serum LYZ (2.11 vs. 1.42 μg/mL C) and IgM (26.28 vs. 21.42 mg/mL C); ↑ *TNF-α*, *IL-1β* and *IL-8* expression; ↓ mortality after *F. oxysporum* challenge (~35% vs. ~65% C). Mortality was also ~35% at 1.0 g/kg and ~50% at 0.5 g/kg, while immune responses increased with dose.	[395]
cMOS MOS MOS + cMOS	Greater amberjack (*Seriola dumerili*)	5 g/kg MOS; 2 g/kg cMOS; 5 g/kg MOS + 2 g/kg cMOS; 90 days	2 g/kg cMOS: ↑ serum bactericidal activity (8.27 vs. 4.89% C); ↓ *Neobenedenia girellae* parasite length (3.32 vs. 4.44 mm C) and density (0.015 vs. 0.101 parasites/cm^2^ C). MOS alone had no significant antiparasitic effect, and the combination provided no additional benefit.	[396]

Note: ↑: increased; ↓: decreased; IFN: interferon; Ig: immunoglobulins; ISG: interferon-stimulated gene; LYZ: lysozyme; PI: phagocytic index; PR: phagocytic ratio; WBC: white blood cells; NK: natural killer cells; ^a^ Fungal abundance is expressed as log *V. botryosa* gene equivalents per μg of spleen.

**Table 11:** Effects of prebiotics in attenuating various stress factors in farmed fish.

Prebiotic	Fish Species	Dose and Duration of Administration	Stress Factor	Main Effect	Reference
Inulin	Nile tilapia (*Oreochromis niloticus*)	0.2%, 0.4%, and 0.8% of the feed; 8 weeks	High salinity: 16 psu	0.4% inulin under 16 psu: restored SOD, CAT, and GST activities toward freshwater levels (SOD: ~175 vs. ~225; CAT: ~10 vs. ~4; GST: ~90 vs. ~30 U/mg protein in the 16-psu C). The 0.2% and 0.8% doses were less effective.	[398]
MOS	Red seabream (*Pagrus major*)	0.5; 1; 1.5 and 2 g/kg feed; 60 days	Low salinity: seawater diluted 1:1 with fresh water	2 g/kg MOS: ↑ tolerance to low-salinity stress (LT_50_: ~102 vs. ~72 min C). The 1 and 1.5 g/kg doses also significantly increased LT_50_, whereas 0.5 g/kg did not.	[399]
MOS	Common carp (*Cyprinus carpio*)	0.2% of the feed; 30 days	Ammonia: 0.15 mg/L NH_3_	↓ cortisol (31.06 vs. 71.36 ng/mL C) and glucose (8.42 vs. 18.73 mmol/L C); restored antioxidant enzyme activities toward control levels and reduced ammonia-induced tissue damage.	[400]
XOS	Hybrid Grouper (*Epinephelus fuscoguttatus* ♀ × *E. lanceolatus* ♂)	0.05% of the feed; 4 weeks	Total ammonia: 1.4 mg/L; 96 h	↑ survival after 96 h ammonia stress (92.5% vs. 52.5% C); ↑ serum GSH-Px and lysozyme activities and ↓ intestinal damage.	[401]
MOS	Gilt-head bream (*Sparus aurata*)	4 g/kg feed; 8 weeks	High temperature: 34 °C; 10 days	↓ cortisol (~12 vs. ~35 mg/dL C), ↑ lysozyme activity (~680 vs. ~350 ng/L C), and ↓ intestinal *il-1β* and *tnf-α* expression after heat stress; survival was not significantly affected.	[402]
MOS + β-glucan (Beta-MOS^®^)	Nile tilapia (*Oreochromis niloticus*)	0.3% of the feed; 60 days	Lead acetate: 10 mg/L	↓ cortisol (129.59 vs. 181.36 ng/L in males; 126.53 vs. 181.36 ng/L in females) and gonadal MDA (7.03 vs. 8.52 in males; 9.47 vs. 11.32 in females); improved gonadal histology under Pb exposure.	[403]
GMOS	European seabass (*Dicentrarchus labrax)*	0.5% of the feed; 9 weeks	High-density crowding: 35 kg fish/m^3^, for 7 days	↓ plasma cortisol 2 h after crowding + *V. anguillarum* challenge (254.29 vs. 611.29 ng/mL C) and ↓ gill *nfκβ2* and *gr* expression; ↑ survival (53% vs. 40% C).	[404]
MOS	Nile tilapia (*Oreochromis niloticus*)	1% of the feed; 30 days	Chlorpyrifos: 15 µg/L	↑ survival under CPF exposure (96.67% vs. 83.33% CPF); ↑ hepatic *GPx* (~2.6 vs. ~0.7) and *CAT* (~2.1 vs. ~1.0) expression; reduced gill, intestinal, and hepatopancreatic lesions.	[405]
Chitosan	Nile tilapia (*Oreochromis niloticus*)*—GIFT*	0.5%, 1%, 1.5%, and 2% of the feed; 60 days	Cadmium: 0.2 mg/L Cd^2+^	At 1.5% chitosan: ↑ serum SOD (24.48 vs. 12.04 U/mL C) and CAT (11.99 vs. 6.44 U/mL C); At 2%: ↓ hepatic MDA (0.43 vs. 1.47 nmol/mg protein C) and gill MDA (1.39 vs. 3.40 nmol/mg protein C). Antioxidant responses varied among doses, with no single level producing the strongest effect for all parameters.	[406]
β-glucan from *Saccharomyces cerevisiae*	Nile tilapia (*Oreochromis niloticus*)	0.1% of the feed; 60 days	Diazinon: 0.28 mg/L in water	↑ survival (~67% vs. 40% DZN control); ↓ hepatic MDA (~10 vs. ~24 nmol/g tissue) and ↑ hepatic SOD (~300 vs. ~185 U/g tissue); ↓ hepatic diazinon residues (0.097 vs. 0.129 ng/g tissue)	[407]

Note: ↑: increased; ↓: decreased; C: Control; MDA: malondialdehyde; SOD: superoxide dismutase; GPx: glutathione peroxidase; psu: practical salinity units; CAT: catalase; GST: glutathione-S-transferase; LT_50_: lethal time to 50% mortality.

## Data Availability

The original contributions presented in this study are included in the article. Further inquiries can be directed to the corresponding authors.
